# Challenges and opportunities for chiral covalent organic frameworks

**DOI:** 10.1039/d2sc02436e

**Published:** 2022-07-20

**Authors:** Xing Kang, Emily R. Stephens, Benjamin M. Spector-Watts, Ziping Li, Yan Liu, Lujia Liu, Yong Cui

**Affiliations:** School of Chemistry and Chemical Engineering, Frontiers Science Center for Transformative Molecules and State Key Laboratory of Metal Matrix Composites, Shanghai Jiao Tong University Shanghai 200240 China yongcui@sjtu.edu.cn; MacDiarmid Institute for Advanced Materials and Nanotechnology, School of Chemical and Physical Sciences, Victoria University of Wellington Wellington 6012 New Zealand luke.liu@vuw.ac.nz; College of Biological, Chemical Sciences and Engineering, Jiaxing University Jiaxing Zhejiang 314001 China

## Abstract

As highly versatile crystalline porous materials, covalent organic frameworks (COFs) have emerged as an ideal platform for developing novel functional materials, attributed to their precise tunability of structure and functionality. Introducing chiral functional units into frameworks produces chiral COFs (CCOFs) with chiral superiorities through chirality conservation and conversion processes. This review summarises recent research progress in CCOFs, including synthetic methods, chiroptical characterisations, and their applications in asymmetric catalysis, chiral separation, and enantioselective recognition and sensing. Challenges and limitations are discussed to uncover future opportunities in CCOF research.

## Abstract 2

As highly versatile crystalline porous materials, covalent organic frameworks (COFs) have emerged as an ideal platform for developing novel functional materials, attributed to their precise tunability of structure and functionality.

## Graphical abstract

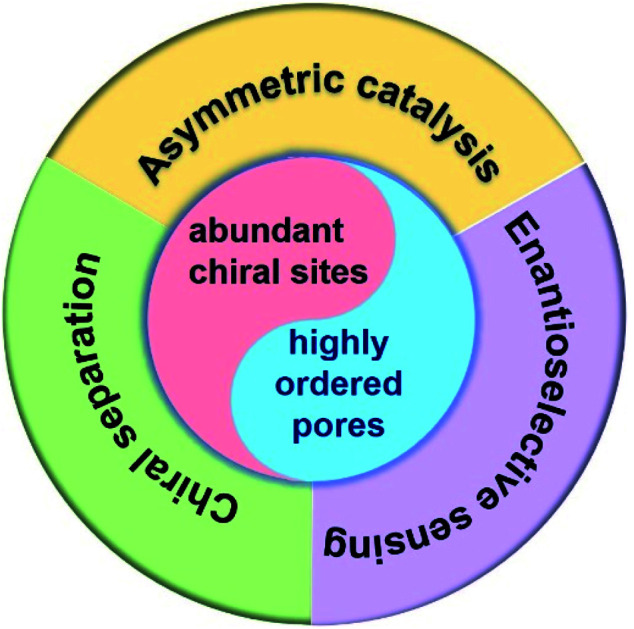

## 1 Introduction

Chirality, a phenomenon that an object that cannot coincide perfectly with its mirror image, is ubiquitous at length scales ranging from neutrinos to spiral galaxies.^1^ In stereochemistry, a chiral molecule and its mirror-image counterpart (a pair of enantiomers) exert different influences on plane-polarised light due to their opposite spatial arrangements of atoms. In addition, different enantiomers exhibit specific physiological activities and chemical selectivities.^2^ For instance, *R*-thalidomide is a prominent sedative to relieve nausea, whereas its mirror counterpart, *S*-thalidomide, is a teratogen that causes congenital disabilities. Their different activities were unknown in the late 1950s, and the racemic mixture (1 : 1 *R*- and *S*-enantiomers) was used to treat morning sickness during the pregnancy and incurred birth defects. This tragedy accelerated the development of enantiomerically pure therapeutics that do not contain the opposite enantiomer. With the further study of optical activities, chiral substances are rapidly developing in pharmaceutical, agricultural, fine chemical, and optoelectronic industries. Preparing optically pure compounds has become an increasingly important research focus.^3^

In recent years, nanoporous materials research has been accelerating partly due to a wide range of applications in storage, separation, catalysis, and optoelectronics.^4^ These materials are highly versatile and can be easily functionalised. Especially, crystalline porous materials, including metal–organic frameworks (MOFs),^5^ covalent organic frameworks (COFs),^6^ hydrogen-bonded organic frameworks (HOFs),^7^ and molecular cages (MCs)^8^ featuring periodic, unambiguous structures and often exhibit permanent porosity. Hence, introducing chirality into these crystalline materials enables applications related to asymmetric expression.^5*e*,7*c*,8*d*^ Moreover, using techniques such as X-ray or electron diffraction, one can perform fundamental studies to delineate structure–property relationships.^9^

**Fig. 1:**
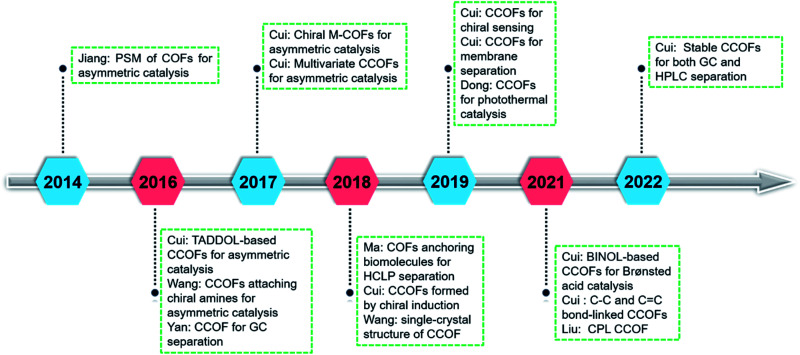
Selected research milestones of chiral covalent organic frameworks.

COFs are synthesised by linking organic monomers into predesigned two- or three-dimensional networks through covalent bonds.^6^ COFs have emerged as a class of crystalline porous materials with potential applications in gas separation and storage,^10^ optoelectronics,^11^ catalysis,^12^ and drug delivery.^13^ The porosity and functionalities of COFs are highly tunable and controllable through judicious selection of organic building blocks. In particular, incorporating chiral functional groups into COFs results in superior chiral functionalities through chirality conservation and conversion processes.^9*b*,14^ For example, molecular chirality can be transferred into the reticular framework through chiral amplification.^15,16^ Moreover, integrating the chirality into a confined network void space can improve enantioselectivity in asymmetric transformations.^15^ The first chiral covalent organic framework (CCOF) and its asymmetric catalysis were reported in 2014 by Jiang and co-workers.^17^ Since then, CCOF research has delivered several milestones (Fig. 1). Chiral expression of CCOFs is regulated at the molecular level through systematic and precise adjustment of structure and functionalisation. However, constructing CCOFs remains challenging owing to the reasons detailed later. As such, the research into chiral COFs is still in the development stage. This review will introduce the research progress of CCOFs, including synthesis, characterisation, and application. In addition, we will discuss the shortcomings of CCOFs and opportunities for future development.

## 2 Design and synthesis of CCOFs

CCOFs with uniform chiral active sites showed fascinating prospects for asymmetric catalysis, chiral recognition, and enantioselective separation. As such, CCOFs have become an important research focus in chiral porous materials. To improve chiral performance, one can fine-tune the chiral environment of the COFs by controlling the chemistry of chiral active sites.^9*a*,18^ While enantiomerically pure building units can only crystallise in 65 Sohncke space groups, most common network topologies for reticular solids (including zeolites, inorganic solids, and MOFs) are observed in more symmetrical space groups. As such, it is reasonable to envision that CCOFs can only be correctly modelled in Sohncke space groups while meeting reticular chemistry requirements. To date, there are three well-established synthetic strategies to construct CCOFs: (1) direct synthesis: using optically pure monomers as cross-linking building units to directly synthesise CCOFs;^19–25^ (2) post-synthetic modification (PSM): reacting achiral parent COFs with chiral molecules to engender chirality *via* the post-synthetic modifications;^26–29^ (3) chiral induction synthesis: using chiral inducing agents to synthesise CCOFs solely from achiral components.^30–32^ This section will review these three synthetic methods for CCOFs.

### 2.1 Direct synthesis

**Fig. 2:**
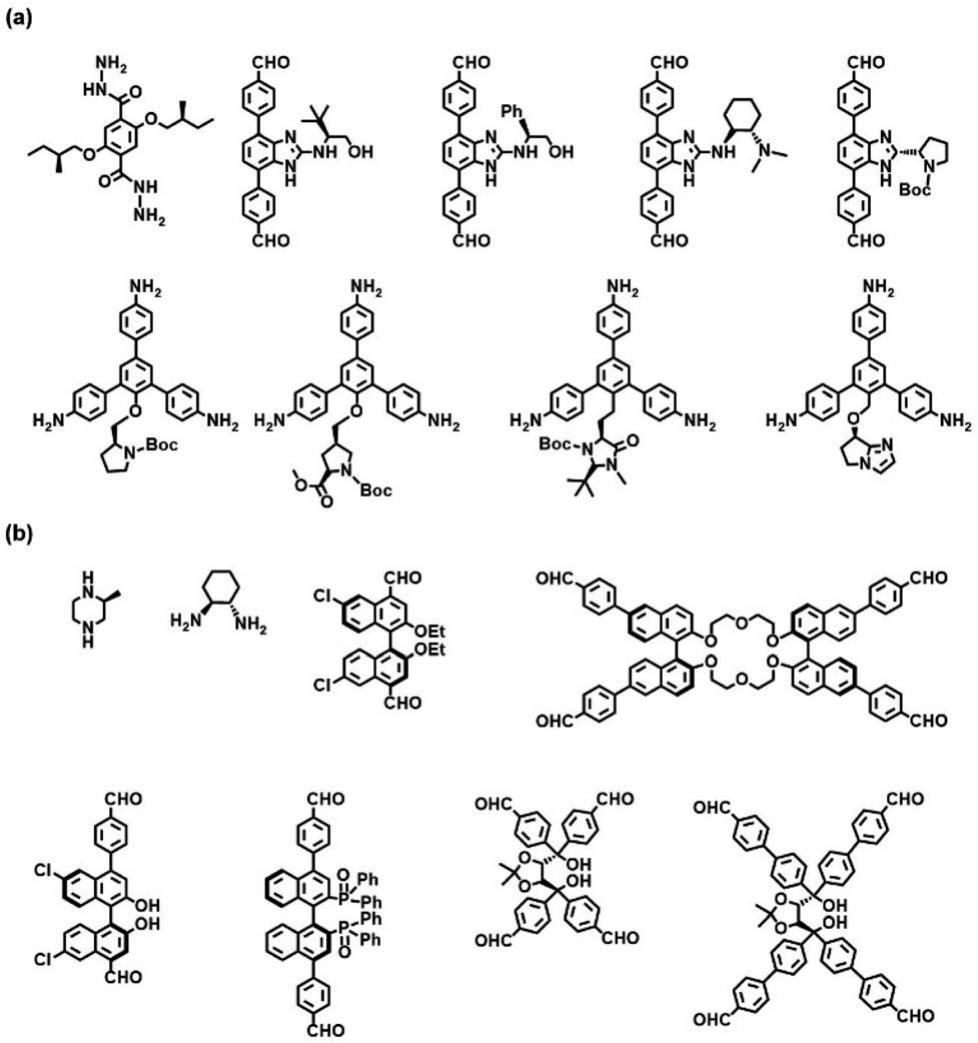
Chiral monomers reported for the construction of CCOFs *via* the direct synthesis: (a) the achiral skeleton monomers and (b) the chiral skeleton monomers.

Direct synthesis is a classical “bottom-up” approach to constructing predesigned homochiral COFs through the rational polymerization of enantiopure monomers according to the design principles of reticular chemistry. Inherent chirality of building blocks is transmitted to the overall framework through chiral conservation and maintains the absolute configuration of the resulting homochiral frameworks. This strategy is expected to evenly distribute chiral sites throughout the framework. There are two types of chiral monomers: (1) chiral skeleton monomers (Fig. 2b), and (2) chiral auxiliary monomers (Fig. 2a). Chiral skeleton monomers have chiral skeletons and often produce CCOFs with higher porosity and larger open channels, whereas chiral auxiliary monomers feature achiral skeletons and chiral functional groups. Chiral auxiliary monomers are used to directly synthesise CCOFs with structures analogous to those obtained by PSM.

**Fig. 3:**
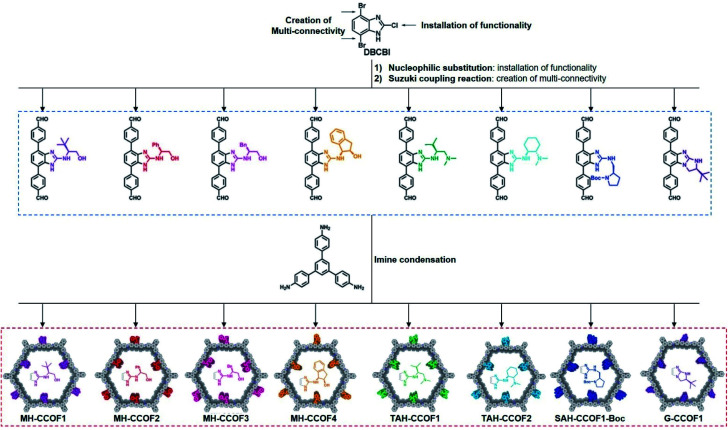
Construction of a series of CCOFs by the divergent strategy. Adapted from ref. 33. Copyright 2019 Wiley.

Wang *et al.* first reported the direct synthesis of CCOFs using enantiopure monomers. These 2D mesoporous chiral frameworks feature high crystallinity and are synthesised from rigid scaffold monomers with chiral pyrrolidine auxiliaries.^19^ Inspired by this work, the same group designed the 4,7-dibromo-2-chloro-1H-benzo[*d*]imidazole (DBCBI) as a platform molecule to immobilise a variety of chiral functional groups through nucleophilic substitutions (Fig. 3).^33^ This “bottom-up”, divergent synthesis afforded eight isoreticular CCOFs, each exhibiting distinct chiral functionality. Such high-throughput CCOF synthesis offered eight heterogeneous catalysts for asymmetric amination of β-ketoesters. In addition, Yan *et al.* developed a *C*_3_-symmetrical scaffold monomer to attach linear chiral carboxylic acids. Direct synthesis of this *C*_3_-symmetrical monomer with linear monomers afforded 2D CCOFs with eclipsed sheets for high-enantioselective chiral separations.^20^ Furthermore, Zhang *et al.* prepared hydrazone-linked homochiral frameworks using 1,3,5-benzene-tricarboxaldehyde and linear monomers featuring chiral chain substituents. These CCOFs exhibited high porosity, excellent chemical stability, and high resolution for separating enantiomers.^34^ During the same period, the Cui group developed a multivariate strategy to synthesise a series of 2D homochiral mesoporous COFs with high crystallinity and stability for asymmetric catalysis. These COFs are synthesised through three-component condensation of triamines and two dialdehydes with and without chiral substituents.^35^

**Fig. 4:**
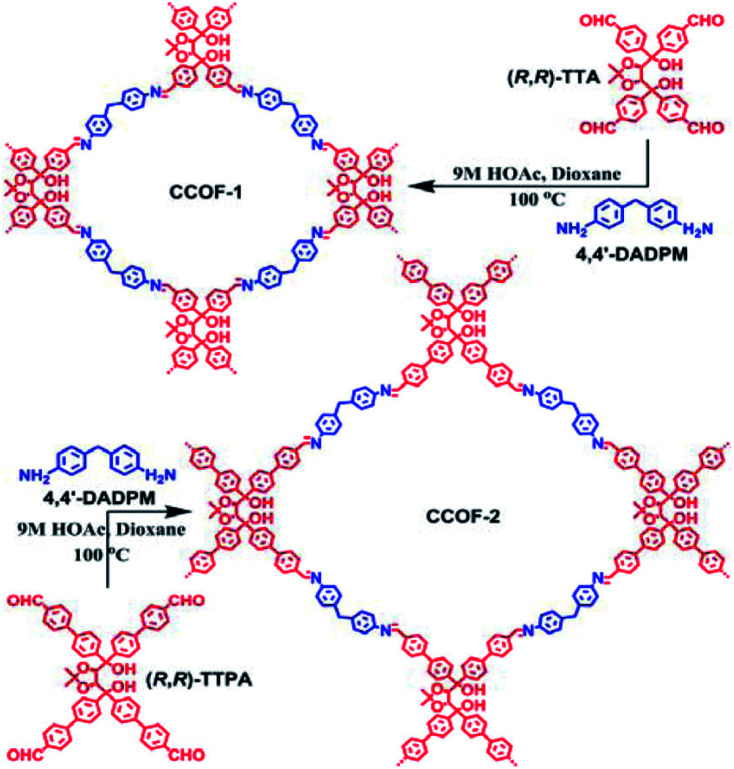
Synthesis of 2D CCOFs from enantiopure TADDOL-derived tetraaldehydes. Adapted from ref. 23. Copyright 2016 the American Chemical Society.

**Fig. 5:**
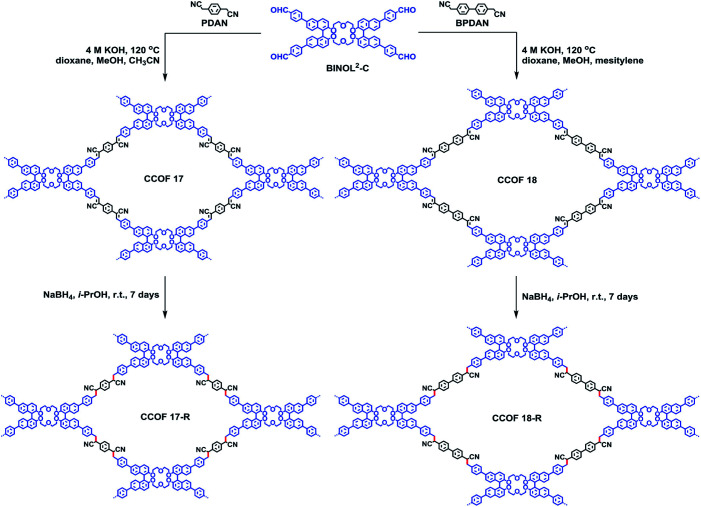
Construction of CCOFs with enantiopure dibinaphthyl-22-crown-6 unites. Adapted from ref. 24. Copyright 2021 the American Chemical Society.

The examples above showcased the synthetic development to append chiral functionalities onto achiral framework skeletons. To further unleash materials' potential for chiral expression, one can envision improving synergy between chiral functionalities and framework skeletons. Towards this end, Cui *et al.* synthesised two COF monomers with an enantiopure skeleton. The obtained enantiopure TADDOL-derived tetraaldehydes react with a diamine linker to form 2D layered CCOFs with high crystallinity and permanent porosity (Fig. 4).^23^ Whereafter, the Cui group tactfully designed and synthesised a variety of enantiopure skeleton monomers to diversify the structure and functionality of CCOFs.^15,24,36–38^ For instance, two isostructural 2D Zn(salen)-based CCOFs with AA stacking hexagonal grid network were constructed *via* imine-condensations of chiral 1,2-diaminocyclohexane and *C*_3_-symmetric trisalicylaldehydes in the presence of Zn^2+^.^36^ These metallo-CCOFs are subsequently metal-exchanged Mn^2+^, Fe^2+^, Cr^3+^, Co^2+^, and V^4+^ to generate a family of metallosalen-based CCOFs. Upon post-synthetic metal exchange, these metallo-CCOFs maintained structural integrity and crystallinity compared to the parent frameworks. As chiral salen ligands are well-known as privileged ligands for asymmetric catalysis, the resultant metallosalen-based CCOFs exhibited excellent enantioselectivities towards catalysing several reactions, with the highest enantiomeric excess (e.e.) of 97%. Similarly, the same group reported two 3D CCOFs with interpenetrated diamond (dia) networks by polymerizing enantiopure BINOL dialdehydes and tetrahedral tetraamine.^15^ Both materials showed superior activities in asymmetric catalysis compared to their homogeneous counterparts. Another example reported by the Cui group is the direct synthesis of 2D olefin-based CCOFs through Knoevenagel polycondensation of diacetonitriles and an enantiopure dibinaph-thyl-22-crown-6 monomer. Subsequent post-synthetic reduction produced C–C bond-linked CCOFs with improved quantum yields and fluorescence lifetimes (Fig. 5).^24^ Additionally, Dong *et al.* synthesised a chiral skeleton COF using *S*-(+)-2-methylpiperazine and cyanuric chloride. They subsequently loaded Pd nanoparticles by impregnating Pd(NO_3_)_2_ followed by NaBH_4_ reduction. This produced a Pd@CCOF composite for catalysing Henry and reductive Heck reactions with high yield and excellent stereoselectivity.^25^ Recently, Liu and co-authors condensed an axially chiral BINAPO linker with 1,3,5-benzene-triacetonitrile to afford a chiroptical CCOF, which demonstrated the amplification of the chiroptical performance in comparison with the homogeneous counterparts.^16^

In summary, direct synthesis produces materials with chiral sites uniformly distributed throughout the framework. An interesting observation is that chiral skeleton monomers are often associated with higher materials porosity and larger pore channels. While this method has attracted wide attention and promoted the development of CCOFs, challenges and opportunities remain as follows:

(1) Chiral monomer skeletons are only compatible with three crystallographic symmetry elements: rotational axis, screw axis, and translation. These symmetry operations generate counterparts that maintain the same absolute configuration. All other symmetry elements, *i.e.* inversion centre, rotoinversion axis, mirror plane, and glide plane are not compatible because they generate counterparts with the opposite chirality. Since there are 65 space groups (Sohncke space groups) that consist only of chiral-compatible symmetry elements, it is reasonable to argue that chiral skeleton COFs should be modelled in one of the Sohncke space groups.^5*e*,9*b*^ On the other hand, COF structures are designed and modelled according to the reticular chemistry principles. A typical process involves (i) choosing a target topology, (ii) designing monomers with geometries and connectivities matching the vertices and edges for the topology, and (iii) building COF models using the space group and key coordinates from the Reticular Structure Database.^39^ While most common network topologies for crystalline solids (zeolite, MOFs, minerals, and inorganic solids) are observed in highly symmetrical space groups, there are only very few topologies observed in Sohncke space groups. The challenge and opportunities involve using these chiral topologies (although less common) to synthesise CCOFs. For example, smt topology is observed in *P*6_5_ and could be used as a blueprint for CCOFs.^40^ In addition, developing unknown chiral topologies can vastly open the possibilities for CCOFs.

(2) Synthesising enantiomerically pure chiral skeleton monomers are nontrivial and need to be geometrically compatible with reticular chemistry concepts. An opportunity may involve taking advantage of naturally-abundant advanced intermediates to synthesise chiral skeleton monomers.

(3) COFs are often synthesised at high temperatures that might risk the racemisation of enantiomerically pure monomers. New synthetic methods might be desirable, such as room-temperature synthesis.^41^

### 2.2 Post-synthetic modification

In contrast to direct synthesis, post-synthetic modification (PSM) introduces chiral substituents after synthesising COFs. PSM accurately pinpoints chiral functionalities at the framework anchor points, and often maintains the overall framework structure.^42^ As such, PSM has become a highly efficient and practical method to regulate the structures and functionalities of COFs. Particularly, chiral moieties were immobilised onto the achiral COF skeletons. Here achiral COFs with appropriate functional groups are subsequently functionalised with chiral substituents to form CCOFs.

**Fig. 6:**
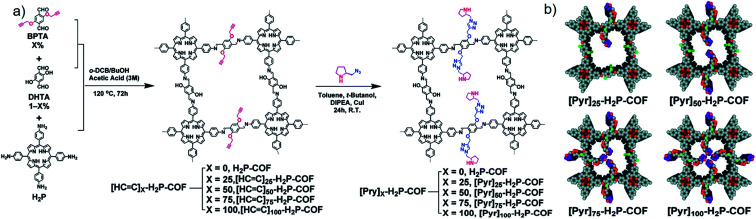
Construction of 2D CCOFs *via* alkyne–azide click reaction: (a) the synthetic route of [Pyr]_*x*_–H_2_P–COFs, (b) the structural representation of [Pyr]_*x*_–H_2_P–COFs with different amounts of chiral sites. Adapted from ref. 17. Copyright 2014 the Royal Society of Chemistry.

In 2014, Jiang and co-authors utilised a reasonable post-synthetic modification to construct CCOFs ([Pyr]_*x*_–H_2_P–COFs, *x* = 0, 25, 75, 100) by anchoring optically pure pyrrolidine on an achiral COF skeleton through the alkyne–azide click reaction (Fig. 6).^17^ BET surface areas and pore sizes of the obtained CCOFs decreased compared to the parent COFs, whereas crystallinity was maintained after PSM. Interestingly, CCOFs displayed improved activity in the asymmetric Michael addition reactions by controlling the loading of organocatalytic sites on the pore walls while retaining enantioselectivity.^17^ Using the same click-reaction, the Jiang group post-synthetically introduced (*S*)-pyrrolidine into an achiral 2D COF at a range of loadings.^26^ The resulting CCOFs exhibit excellent stability, porosity, crystallinity, and an over 90% enantioselectivity (e.e.) towards catalytic Michael addition of β-nitrostyrenes under aqueous conditions. Whereafter, Zhang *et al.* developed chiral ionic COFs (CCLSM-1) with high crystallinity by anchoring the chiral prolinol derivative onto the achiral pyridine-based COF skeleton in an ordered manner.^28^ The resulting ionic CCOF exhibited high catalytic activity and enantioselectivity in asymmetric Henry reactions.

**Fig. 7:**
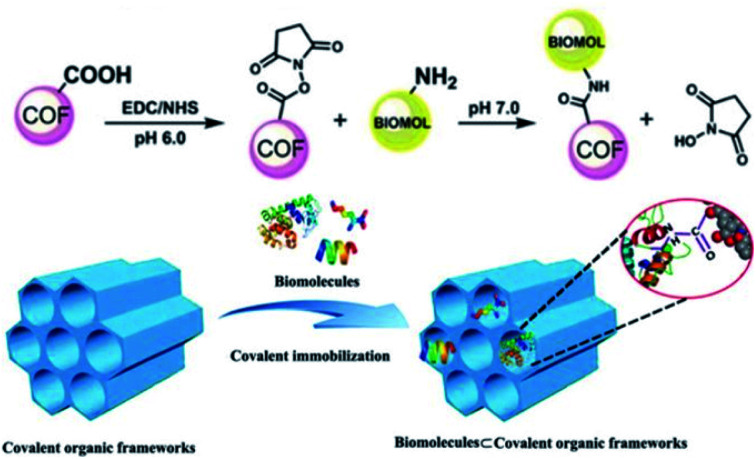
Covalently immobilizing biomolecules into COFs. Adapted from ref. 44. Copyright 2018 Wiley.

In addition to optically active organic small molecules, a range of optically pure functional components, including polymer chains, peptides, and enzymes, are implanted into the pore walls of achiral COFs *via* PSM, affording CCOFs with a wide range of functionalities.^26,43–45^ Based on enantioselective recognition of biomolecules, Ma *et al.* implanted chiral biomolecules including lysozyme, tripeptide, and lysine into achiral mesoporous COFs. The chemistry that links the framework with the biomolecules is the –COOH groups on the framework, and the –NH_2_ groups on the biomolecules, forming amide bonds (Fig. 7).^44^ The resulting biomolecule⊂COFs are used for chiral separations. Biomolecule-containing CCOFs have the same frame structure as the parent COFs, but with reduced surface area and pore size. Interestingly, biomolecule⊂COFs exhibited high chiral separation efficiency for various enantiomers in high-performance liquid chromatography (HPLC). In addition, Ma *et al.* modified optically pure lipase into the mesoporous COFs to generate chiral biocomposites in phosphate buffer solution. Notably, these biocomposites enhanced the stability and robustness of the lipase PS, while the lipase-containing CCOFs exhibited enhanced activity compared to the amorphous analogues.^27^

**Fig. 8:**
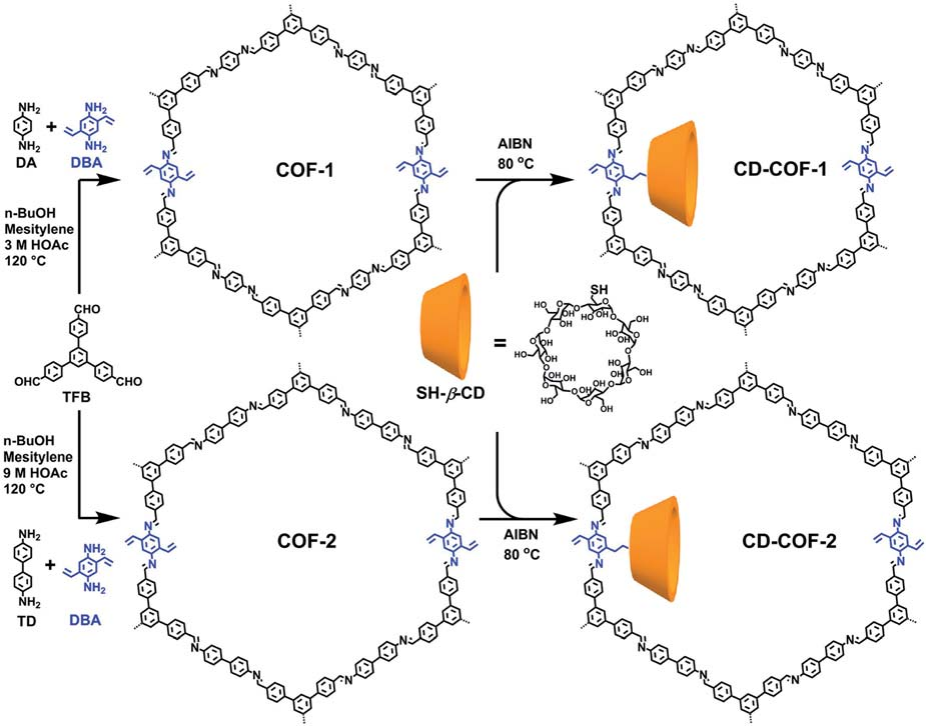
The introduction of chiral β-CD into achiral COFs *via* thiol–ene click reactions. Adapted from ref. 45. Copyright 2019 the American Chemical Society.

Cyclodextrins are common chiral selectors with enantioselectivity. Toward this end, Cui *et al.* prepared CCOFs by introducing chiral β-cyclodextrin (β-CD) into the hexagonal channels of 2D COFs *via* a thiol–ene click reaction (Fig. 8).^45^ Upon PSM, the as-prepared CCOFs maintained the same crystal structure of the parent COFs. As expected, both surface area and pore size were reduced after PSM. Nevertheless, CCOFs with low β-CD loading exhibited high enantiotopic discrimination towards amino acids. Recently, Ji *et al.* synthesised optically pure COF post-synthetically by linking optically pure heptakis(6-amino-6-deoxy)-β-CD (Am7CD) with a carboxyl-functionalised COF, TpBD–3COOH.^29^ The resulting CCOF, TpBD-Am7CD, is more selective for adsorbing amino acid compared to the parent material, suggesting that the chiral CD functionality provides a chiral environment.

From the above research progress, it is clear that PSM provides a simple and effective method for synthesising the functionalised CCOFs. However, some shortcomings remain. For example, while many reports showed that optimal chiral separation/catalysis performances are associated with low loadings of chiral functionalities, spatial distributions of these functional groups are not clear. A method to control spatial distribution, whether homogenous throughout the framework, or deliberately introducing heterogeneity in a managed fashion, will be desirable. Additionally, upon post-synthetically introducing chiral functionalities, COF porosity drops inevitably. While lower porosity is not necessarily undesirable, a strategy to engineer pore metrics, including pore-size distribution, and pore-limiting diameter for balancing mass transport and molecular sieving, is worth developing.

### 2.3 Chiral induction synthesis

Since the successful embedding of chirality of enantiomeric polysaccharide molecules into achiral molecular systems, chiral induction synthesis has become one of the most attractive methods for synthesising chiral substances using achiral reagents. Chiral induction has been widely used for preparing optically active small molecules, polymers, and supramolecular systems for chiral amplification.^46^ In particular, chiral induction has produced optically pure polymers and empowered these materials with new applications. As a result, chemists have started to use small chiral molecules to induce chirality for synthesising CCOFs using achiral precursors.

**Fig. 9:**
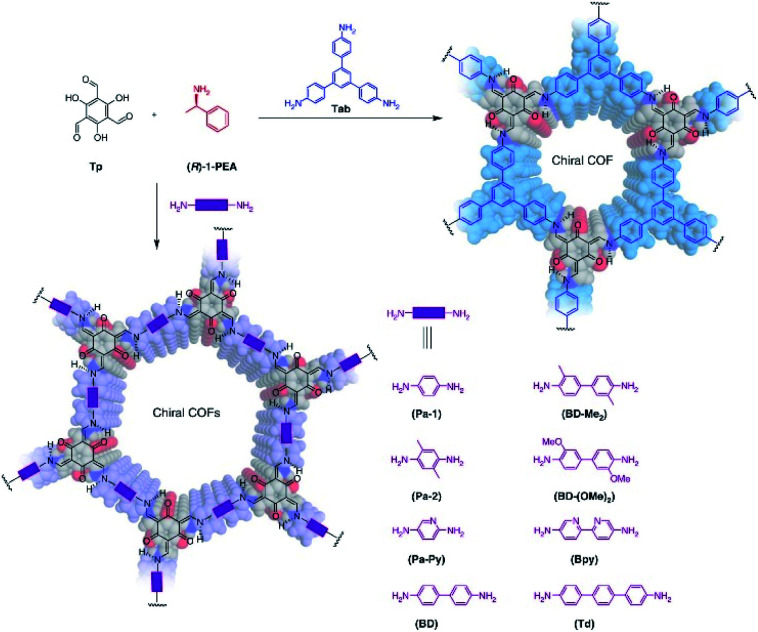
Synthesis of 2D CCOFs from achiral precursors by chiral catalytic induction. Adapted from ref. 30. Copyright 2018 Nature Research.

In 2018, Cui *et al.* first employed the chiral induction method to synthesise a family of CCOFs featuring three-bladed propeller geometry. These CCOFs are condensed from achiral ditopic or tritopic amines and 1,3,5-triformylbenzene in the presence of optically pure 1-phenylethylamine (Fig. 9).^30^ The chirality of the CCOFs originated from the conformational conversion of the TASN derivatives promoted by intramolecular hydrogen bonding during the chiral induction. In addition, CCOF-TpTab exhibited high enantioselectivity for chiral carbohydrates. After post-synthetic metalation with Cu(ii) ions, the metallo-CCOFs exhibited high stereoselectivity in the asymmetric Henry reaction. Subsequently, Dong *et al.* prepared an enantiopure COFs with high crystallinity through polymerisation of achiral dialdehydes, triamines, and terminal aryl alkynes under the catalytic asymmetric induction of Cu(i)/chiral pybox.^31^ The resulting CCOFs are catalytically active towards Michael addition with decent activity and enantioselectivity. In addition, the same group employed an amino-catalysed asymmetric approach to facilitate the stereoselective condensation of tricarbonyl phloroglucinol with different amines or hydrazides to construct a variety of 2D CCOFs that can chelate metal ions and act as heterogeneous catalysts for asymmetric transformations.^47^ Using chiral induction of enantiopure amines, Gu and co-workers synthesised ultrathin 2D CCOFs from achiral precursors for circularly polarized luminescence (CLP).^32^

Although introducing exogenous chiral auxiliaries transfers and amplifies chirality towards achiral materials to produce enantiopure COFs, chiral induction methods for COOFs are still in their infancy and challenges remain. So far, there are only several chiral induction molecules used to synthesise CCOFs. Hence, it is necessary to introduce other chiral inducers, such as circularly polarised light and chiral solvents, to develop synthetic strategies for CCOFs. Furthermore, there is a lack of understanding of the induction mechanism. Fundamental mechanistic studies in chiral induction may improve predictability for future chiral induction synthesis.

## 3 Chiroptical characterisation

Chiral materials have been shown to have diverse applications in biology, medicine, pesticides, polymers, and smart materials.^48^ In recent years, homochiral COFs have attracted much attention in the field of chiral separation and asymmetric catalysis among others, attributed to their unique feature of function and structure. Therefore, it will be valuable to obtain chiral materials with optical activities. It is tremendously important to determine the absolute configuration of enantiopure COFs, which is beneficial to accurately clarify their mechanism of chiral expression including chiral recognition, asymmetric transformation, and enantioselective separation. At present, most COFs are crystalline powders rather than single crystal materials due to their difficulty in crystallization.^49^ The main methods to measure the absolute configuration of CCOFs are the chiroptical method including circular dichroism (CD),^23,30,35^ and circularly polarized luminescence (CPL).^16,32^ This section will review the principles and applications of the absolute configuration determination methods for CCOFs.

### 3.1 Circular dichroism spectra

CD is an effective spectroscopy method for the structural characterisation of chiral systems through the differential absorption of left-right circularly polarized light in the ground state. The principle of CD relies on the molecular absorption coefficient difference between left-rotation light (*ε*_L_) and right-rotation light (*ε*_R_), namely Δ*ε* = *ε*_L_ − *ε*_R_.^50^ The intensity of each transition in CD spectra is proportional to an oscillator and rotational strength, respectively. In addition, the CD spectra of enantiomers rotate light in opposite directions with the same rotational intensity and the same number of absorption peaks. Generally, the CD spectrum is characterised by the UV-vis region where electronic transitions are more active. The UV-vis region can be effectively monitored and recorded using the electronic transitions of the chromophore. Therefore, it is also referred to as electronic circular dichroism (ECD), which can be widely applied to identify the absolute configuration of chiral materials such as MOFs, cages, and supramolecules.^51^

**Fig. 10:**
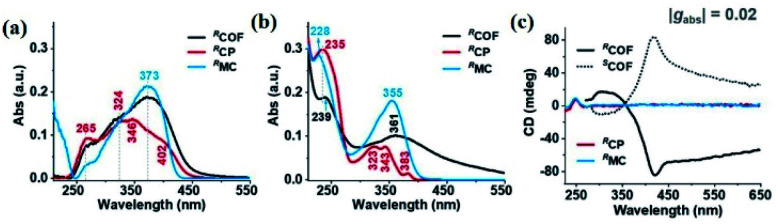
The optical and chiroptical properties of COF. (a) UV/Vis diffuse-reflectance spectra of solid-state COF, CP, and MC. (b) UV/Vis, (c) ECD spectra of COF, CP, and MC. Adapted from ref. 16. Copyright 2021 Wiley.

**Fig. 11:**
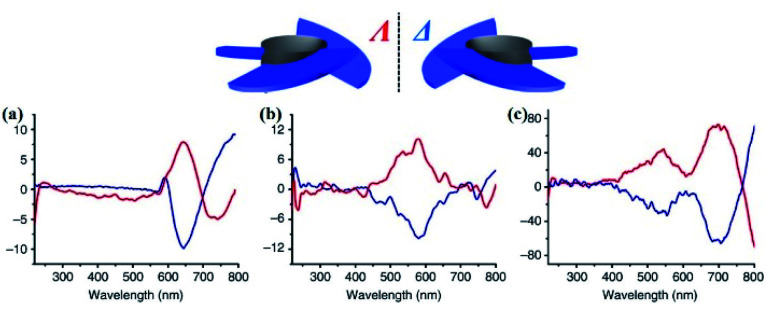
ECD spectra of CCOFs induced by (*R*)-1-PEA (red line) and by (*S*)-1-PEA (blue line). (a) TpPa-1, (b) TpPa-2, (c) TpPa-Py, respectively. Adapted from ref. 30. Copyright 2018 Nature Research.

ECD spectra of the following CCOFs were obtained from chiral building blocks, these chiral materials exhibit the Cotton effect and produce ECD spectra that are mirror images of each other. These mirrored spectra demonstrate that the enantiomeric nature is completely controlled by the chirality of the building monomers in the ground state. The ECD spectra are consistent with their corresponding UV-vis spectra. As shown in Fig. 10(c), the ECD spectra of CCOFs with BINAPO skeletons show a Cotton effect with asymmetry factor (*g*_abs_) up to 0.02. This value is higher compared to the corresponding polymeric analogue (CP), or the molecular model compound (MC), suggesting that stacked COF layers are beneficial to visualising the ground-state chirality.^16^ The ECD response of exfoliated 2D COFs (COF-e) diminished substantially from parent COFs by nearly an order of magnetite, suggesting a lack of efficient dual confinement of the reticular framework and interlayer stacking (Fig. 10). In addition, Cui *et al.* prepared a series of CCOFs constructed from achiral organic precursors *via* chiral catalytic induction. These CCOFs were induced by the presence of enantiomerically pure 1-PEA and displayed the Cotton effect in solid-state ECD spectra. CCOFs with the opposite chiralities were produced to generate either (Λ)- or (Δ)-frameworks, indicating the success of asymmetric induction (Fig. 11).^30^ Optical activities of DTP-COFs and TpPa-1 were confirmed by ECD spectra, implying that chiral-inducing agents catalysed the formation of CCOFs. Zhang *et al.* utilised ECD to confirm the absolute configuration of CCLSM-1, and the optical activity shown after the incorporation of chiral prolinol bromoacetate indicates that chirality has been incorporated into the framework.^28^

Although ECD can be used to study the chiral structural information of CCOFs in the ground state, the ECD spectra of microcrystalline powdered CCOFs generally suffer from the inhomogeneity of the sample preparation. The ECD spectra of CCOFs are unsuitable at higher concentrations and are susceptible to distortion due to absorption flattening (AF) and scattering effects, which affect the accuracy of the structural information of CCOFs. Therefore, it was necessary to prepare a fine and uniform sample for testing, in addition to single crystals. Additionally, the absolute configuration of the resulting CCOFs was currently only qualitatively analysed by ECD, confirming optical activities of CCOFs is still in the preliminary stage. Particularly, the relationship between the chiral spectra and stereochemical/electronic structures of CCOFs will likely be revealed further by the ECD spectra, for example, the effect of anisotropy of CCOFs on ECD and the effect of electronic leaps of chromophores and chiral centres on the *g*_abs_ need to be explored. These researches will contribute to understanding the chiral optical properties of CCOFs and developing late-model CCOFs. Again, when the chromophore of CCOFs is far from the chiral centre, the structural information of the materials studied by ECD spectra will be not reliable. In order to accurately describe the chiral stereochemical structure of the CCOFs in the ground state, it is necessary to combine other CD spectra such as vibrational circular dichroism (VCD), fluorescence circular dichroism (FDCD), and magnetic circular dichroism (MCD) to form complementary information.

### 3.2 Circularly polarized luminescence

CPL defines the differential emission of left- and right-circularly polarized light from chiral luminescent systems. Compared with CD for the ground-state chirality of materials, CPL characterises the configuration and conformation of the chiral luminescent system at the photoexcited state. The use of CPL shows the potential application in photocatalytic asymmetric synthesis, biological probing, 3D displays, polarised laser devices, and chiral sensing. To evaluate the intensity of CPL, the luminescence dissymmetry factor *g*_lum_ is defined as a crucial parameter for the optical activity of chiral materials, owing to the difficulty of characterising absolute emission intensities.^52^ While *g*_lum_ > 0 indicates left-handed polarised light, *g*_lum_ < 0 demonstrates right-handed polarised light. The dextrorotatory circularly polarised light is typically shown between −2 and +2. In general, the construction of CPL materials requires an achiral luminophore in combination with a chiral unit. To date, numerous CPL materials like polymers, organic molecules, inorganic complexes, and supramolecules, have been developed for CPL activity. Recently, COFs have been of particular interest in chiroptical materials with CPL properties, owing to their structural tunability and the fact that they can be functionalised easily.

**Fig. 12:**
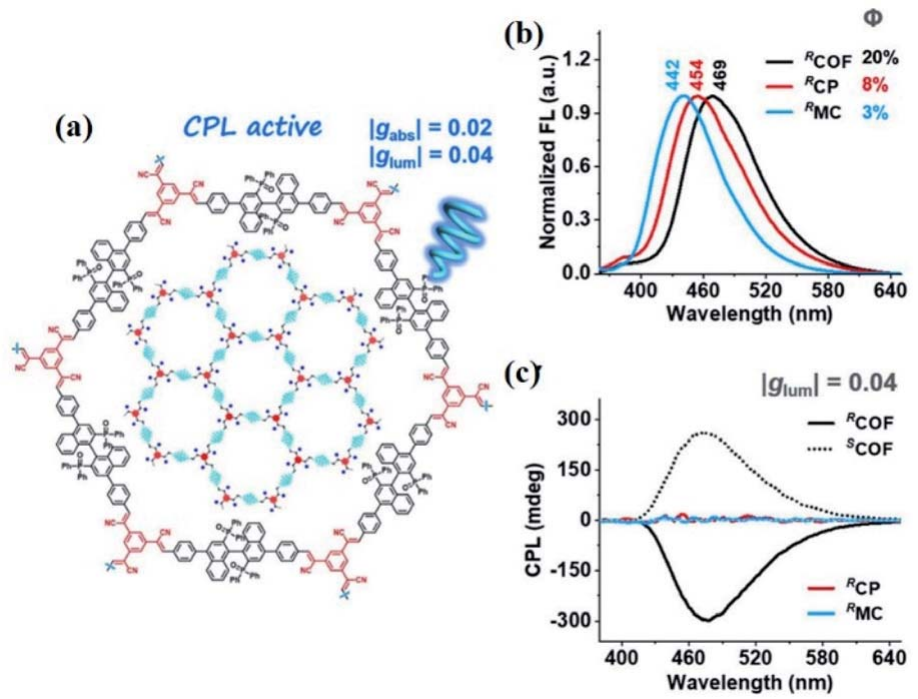
(a) Illustration of the CPL-based COF, (b) FL, and (c) CPL spectra of COF, CP, and MC. Adapted from ref. 16. Copyright 2021 Wiley.

In 2021, Liu *et al.* reported the first CPL-active CCOF constructed from an axial chiral BINAPO derivative. The CCOFs demonstrated stronger CPL activities with the absolute *g*_lum_ up to 0.04 compared to corresponding MC, CP, and COF-e. The intensity of *g*_lum_ illustrates the remarkably magnified chiroptical characteristic of CCOFs in the excited state, attributed to the cooperative effect of the reticular frame and interlayer stacking (Fig. 12).^16^ Subsequently, Gu *et al.* utilised chiral-induced synthesis to successfully fabricate ultrathin luminescent CCOFs nanosheets (NS) with a |*g*_lum_| of the red CPL performance as high as 0.02.^32^ Intriguingly, the green and blue fluorescencing dye molecules were immobilised on the CCOFs NS (CCOF/Dyes) with uncoordinated amino groups. This coordination of the CPL emissive light has been ascribed to the chirality and energy transfer between CCOFs and dye groups *via* the hydrogen-bond interaction. Additionally, the as-prepared CCOFs/Dyes displayed strong chirality and amplified CPL activities with |*g*_lum_| up to ∼0.1, which was ∼5 times stronger than that of corresponding CCOFs NS. More importantly, CCOFs/Dyes NS were suitable to be scattered into a polydimethylsiloxane (PDMS) matrix to acquire transparent and flexible COFs composite films for practical CPL applications.

The aforementioned case studies demonstrate that CPL can be used to determine the absolute configuration of CCOFs. However, there are no fully-fledged methods for the design of CCOFs with CPL performance. Particularly, the construction of the CPL-based CCOFs with high |*g*_lum_| values remains an enormous challenge due to the complex relationship between chirality and fluorescence. Hence, CPL-emissive COFs will need to be a focus in the future development of this field along with the development of novel CCOFs that possess a high quantum yield and CCOFs with high asymmetric factors. Another very useful investigation will highlight the chiral transfer mechanism of the chiral source to the luminophore in CCOFs. The chiral transfer mechanism from the luminophore is of great significance as it produces a greater degree of polarization and in theory, makes the CPL of CCOFs easier to measure. Therefore, challenges and opportunities coexist, and it is expected that the exploration of CPL-based COFs and their assemblies will have important significance and broad application prospects.

## 4 Application of CCOFs

As a new type of chiral material, CCOFs possess highly ordered pores, abundant chiral sites, and uniform distribution of chiral components. These unique features result in an excellent performance in chiral applications by adjusting the structure and functionality. At present, CCOF applications mainly focus on the following three aspects: asymmetric catalysis, chiral separation, and enantioselective sensing. In this section, the applications of CCOFs are described in detail and discussed while the existing problems and limitations regarding synthesis and application are summarised.

### 4.1 Asymmetric catalysis

Asymmetric catalysis generally refers to the use of chiral catalysts to selectively convert prochiral substrates into chiral products with specific configurations. This is achieved by preferentially lowering the activation energy for one diastereomer thus making a product with a specific chirality.^53^ Sharpless *et al.* reported an asymmetric dihydroxylation reaction with high enantioselectivity through the dynamic transformation of the favourable diastereomeric osmaoxetane intermediate in the DHQD–osmium complex catalyzed reaction.^54^ In 2017, List *et al.* reported the enantioselective synthesis of various oxygen heterocycles from lactol acetate and silylated nucleophiles through the formation of the diastereoisomeric oxocarbenium ion complex with the chiral counteranion.^55^ While You *et al.* employed cyclopentadienyl rhodium complexes to promote a stereoselective [4 + 1] annulation reaction of benzamides and alkenes for a range of isoindolinones with excellent regio- and enantioselectivity through an oxidative Heck reaction and an intramolecular enantioselective alkene hydroamination reaction.^56^ The development of numerous homogeneous chiral catalysts with high selectivity and efficiency has made asymmetric synthesis a popular research topic that has subsequently promoted the development of optically pure compounds in the past decade. However, the purification of homogeneous catalysts is cumbersome to separate and not typically recycled, which restricts their application in industry. To overcome these problems, immobilisation of homogeneous catalysts on solid supports provides a feasible solution, that provides significant advantages over homogeneous catalysts, including better recyclability, continuous batch processing, and being more environmentally friendly than non-solid support catalysts.^9^ In addition, heterogeneous catalysts with a unique chiral environment exhibit the confinement effects to promote better stereoselectivity than their homogeneous counterparts in asymmetric catalysis. Hence, the development of heterogeneous catalysts has attracted increasing attention in recent years.

In consideration of their high porosities, well-defined channels, and tunable functionalisation, COFs have emerged as promising solid-state heterogeneous catalysts as they provide many characteristics desirable for a high-performance catalyst. Therefore, COFs provide a perfect platform to develop heterogeneous catalysts, which can improve catalytic performance by adjusting the functioned building units. In particular, CCOFs immobilising chiral homogeneous catalysts can promote asymmetric transformations through the confinement effect of frameworks and stereoselective activation of chiral catalytic sites. Since the first example of CCOFs promoted asymmetric Michael additions, some privileged chiral ligands and organocatalysts have been successfully implanted into COFs. These chemical moieties are typically installed through various suitable methods and have demonstrated excellent enantioselectivity and reusability in numerous asymmetric syntheses.^9*b*,14^ This section will introduce an overview of the state of CCOFs for asymmetric heterogeneous catalysis and discuss some potential applications and limitations.

**Fig. 13:**
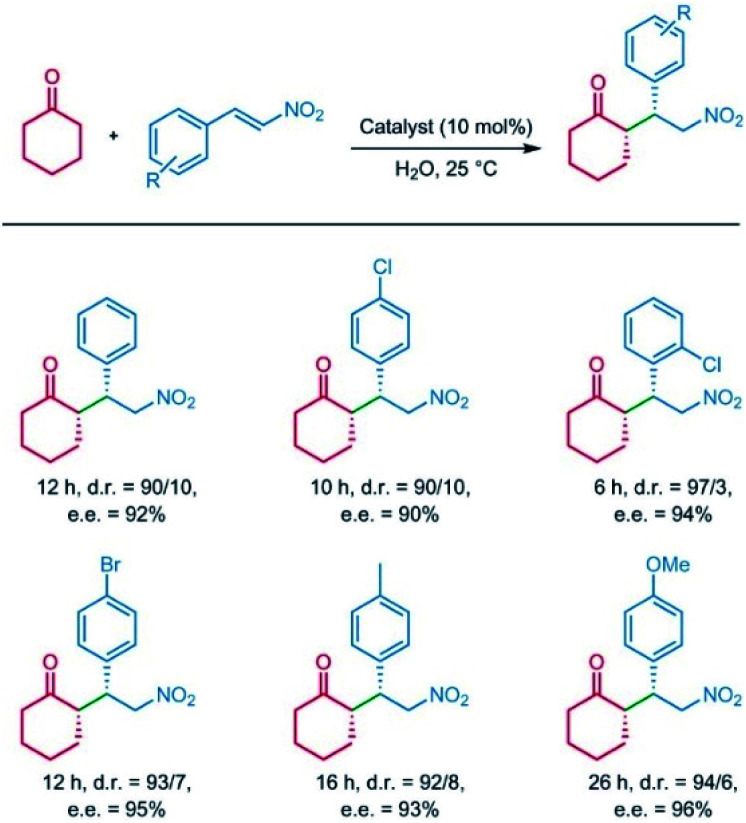
The Michael reactions of β-nitrostyrene promoted by [(*S*)-Py]_0.17_-TPB-DMTP-COF catalyst. Adapted from ref. 26. Copyright 2015 Nature Research.

In 2014, Jiang *et al.* reported a series of CCOFs ([Pyr]_*x*_–H_2_P–COFs, *x* = 0, 25, 75, 100) with different concentrations of chiral organocatalysts that served as chiral heterogeneous catalysts for the asymmetric Michael addition reactions. These CCOFs exhibited decreasing catalytic activity as the concentration of the catalyst increased in the channel, attributed to the blockage of mass transport channels by the organocatalyst.^17^ Notably, [Pyr]_0.25_–H_2_P–COF showed significantly higher catalytic activity than its homogeneous counterpart and amorphous or non-porous catalysts under the optimized condition. This data suggests that the PSM of COFs using chiral moieties significantly enhances catalytic activity. However, a slight decrease in catalytic activity and surface area as measured by N_2_ adsorption/desorption isotherms after recycling may be ascribed to the poor stability of the resulting CCOFs. Considering that the stability of 2D COFs is related to the interlayer interactions, the Jiang group subsequently synthesised a series of high stability CCOFs ([*S*-Py]_*x*_-TPB-DMTP-COFs, *x* = 0.17, 0.34, 0.50) by introducing methoxy groups into the pore walls to enhance interlayer interactions.^26^ This increase in stability is due to inhibition of the interlayer charge repulsion from the polarised C=N bonds in imine-linked COFs by delocalizing the lone pair of the electrons on the methoxy groups over the phenyl rings with positive charge *via* resonance. Additionally, the resulting CCOFs with chiral proline successfully drove the addition of cyclohexanone and β-nitrostyrene derivatives showing high e.e. and dr values of 90–96% and 90/10–97/3, respectively. Interestingly, the [(*S*)-Py]_0.17_-TPB-DMTP-COF as a heterogeneous catalyst afforded higher catalytic activity than the molecular catalyst (*S*)-Py (Fig. 13), presumably due to the open channels of CCOFs in support of enrichment of the reactants from the aqueous for promoting the reaction in the confined framework. Again, the enantioselectivity and diastereoselectivity [(*S*)-Py]_0.17_-TPB-DMTP-COF were comparable to that of (*S*)-Py, which demonstrated that the chiral catalytic sites on the channel walls maintained both enantiocontrol and diastereocontrol in the framework. However, the catalytic activities of [(*S*)-Py]_0.34_-TPB-DMTP-COF and [(*S*)-Py]_0.50_-TPB-DMTP-COF were inferior to that of [(*S*)-Py]_0.17_-TPB-DMTP-COF. Addition under the same reaction conditions further confirmed that excessive organocatalysts anchoring on the channel walls will prevent mass transport through the channels.

Recently, Zhang *et al.* reported the CCLSM-1 with the organic cationic ionic liquids (OCILs) catalysts as a heterogeneous catalyst promoted the asymmetric Henry reactions between nitromethane and benzaldehyde up to 97% yield and 92% e.e., respectively.^28^ In addition, the catalysts can be readily reused and maintain catalytic activity only showing a slight decrease in enantioselectivity after 5 recycles. This demonstrates that OCILs immobilised on CCLSM-1 possess excellent recyclability in an asymmetric catalyst system.

In 2016, Wang *et al.* made LZU-76 through direct synthesis as a heterogeneous chiral catalyst to promote the asymmetric aldol reaction of aromatic aldehydes with acetone.^19^ Compared with post-synthetically modified CCOFs, the chiral catalytic sites of LZU-76 could be evenly distributed in the channel to achieve the maximum content of chiral catalytic sites for transportation of reactants and products. As expected, LZU-76 exhibited high catalytic activity and excellent enantioselectivity (88.4 : 11.6–94.0 : 6.0) under optimized conditions. Interestingly, the enantioselectivity of LZU-76 was comparable to that of the homogeneous counterpart. Moreover, LZU-76 could be recovered and recycled at least three times without loss of enantioselectivity. Inspired by this research, the Wang group developed a two-stage divergent strategy to synthesise a series of 2D CCOFs by introducing different chiral organocatalysts, which provided a key platform for investigating structure–activity relationships by accurately controlling the functionalised CCOFs at the molecular level.^33^ These findings suggest that the activity and stereoselectivity of TAH-CCOF2 as a heterogeneous catalyst for an asymmetric amination reaction were superior to that of other resultant CCOFs under the same conditions, and had no obvious loss of enantioselectivity after being reused seven times.

**Fig. 14:**
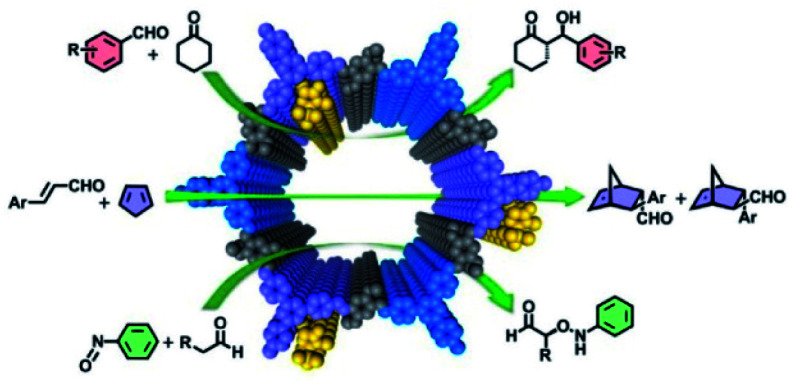
Schematic illustration of a series of asymmetric reactions promoted by the ternary CCOFs. Adapted from ref. 35. Copyright 2017 the American Chemical Society.

In addition, Cui and Liu *et al.* adopted the multivariate strategy to obtain a range of 2D CCOFs including chiral pyrrolidine and imidazolidine catalysts with differing chiral component concentrations for asymmetric organic reactions through direct aldimine condensation (Fig. 14).^35^ Interestingly, the ternary CCOFs showed better crystallinity and stability than the binary CCOFs under harsh conditions by fine-tuning the concentration of organocatalysts attached to the channel walls. The ternary CCOFs functioned well as heterogeneous catalysts and successfully promoted asymmetric catalysis. For instance, DMTA-TPB1/5′ favourably achieved the α-aminooxylation of aldehydes and nitrosobenzene in 75–77% isolated yields with up to a 95% e.e., while DMTA-TPB1/4′ successfully catalysed an aldol reaction between aryl aldehydes and cyclohexanone with 94–95% yield, 86–92% e.e., and 90 : 10 anti/syn ratio, respectively. The catalytic effect of the resultant CCOFs was superior to that of corresponding noncrystalline polymers, while the stereoselectivity and diastereoselectivity of CCOFs matched or surpassed their homogeneous counterparts. Additionally, the CCOF catalysts could be readily recycled and reused without an obvious loss of catalytic performance after 5 runs.

TADDOL derivatives as privileged chiral ligands play an important role in asymmetric catalysis.^57^CCOF-1/2 containing enantiopure TADDOL units promoted the asymmetric addition of aromatic aldehydes with diethylzinc by treatment with Ti(O_*i*_P_r_)_4_ to afford the desired enantiopure secondary alcohols with high conversion (96–99%) and stereoselectivity (74–94% e.e.).^23^ These CCOFs showed enantioselectivities rivaling those of their homogeneous counterparts. Besides, the CCOF catalysts could be recovered and reused for at least four runs without loss of activity and enantioselectivity. However, the periodic structures of CCOF catalysts became severely distorted after five runs due to poor stability.

**Fig. 15:**
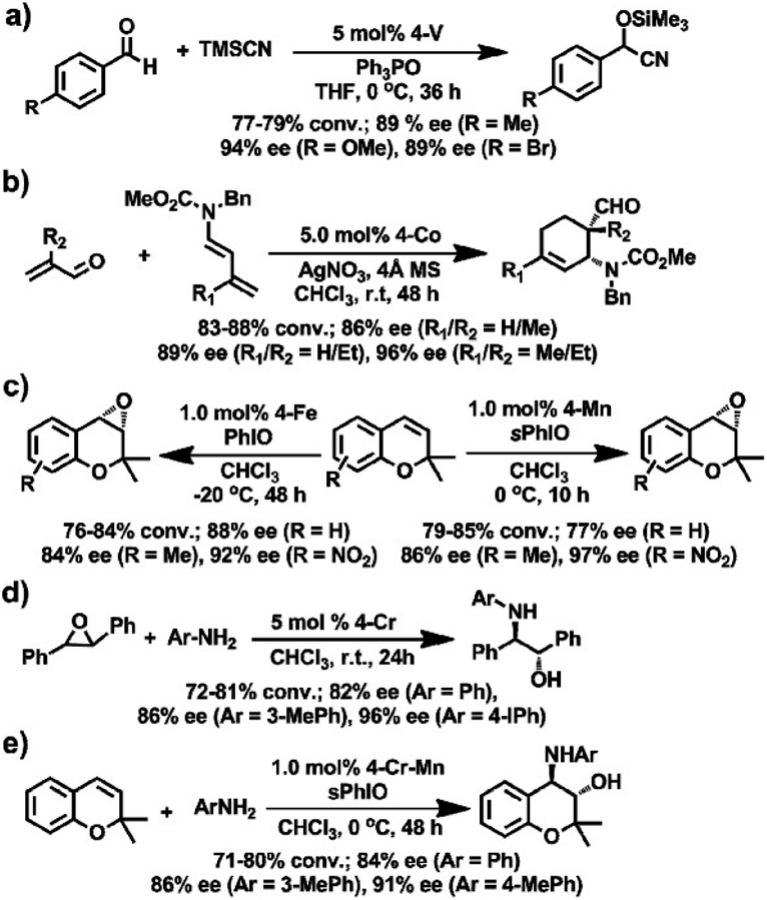
Asymmetric reactions driven by M(salen)-based CCOFs: (a) the cyanation of aldehydes with TMSCN, (b) the DA reaction of butadienes and roleins, (c) the epoxidation of alkenes, (d) the aminolysis reactions, (e) the sequential reactions between the epoxidation and the ring opening of epoxide. Adapted from ref. 36. Copyright 2017 the American Chemical Society.

Subsequently, a series of the M(salen)-based CCOFs (4-M, M^n+^ = Cr^2+^, Co^2+^, Mn^2+^, Fe^2+^, V^4+^) were synthesised by ion exchange treatment of CCOFs with Zn(salen) and used as heterogeneous catalysts for several asymmetric transformations including the asymmetric cyanation of aldehydes, Diels–Alder (DA) reactions, alkene epoxidations, epoxide ring-openings, and aminations (Fig. 15).^36^ For instance, CCOF-4-V achieved cyanation of aldehydes with TMSCN in 77–79% conversion and 89–94% e.e. (Fig. 15a). CCOF-4-Co could afford the desired cycloadducts with 86–96% e.e. in asymmetric DA reactions while CCOF-4-Cr promoted the aminolysis of the epoxides to obtain amino alcohols with 82–96% e.e. (Fig. 15b and d). Noting, a COF implanted with a mixture of metals, CCOF-4-Cr-Mn catalyzed the epoxidation of alkenes and the ring-opening of epoxides to realise the sequential reactions with up to 91% e.e. (Fig. 15e), which provides a valuable strategy for the preparation of complex compounds with excellent enantiopure. The enantioselectivities of CCOFs with M(salen) are comparable to those of the corresponding homogeneous counterpart and other M(salen)-based heterogeneous catalysts. Again, a version of CCOF4-M can be reused for at least five runs without significant loss of activity and enantioselectivity.

**Fig. 16:**
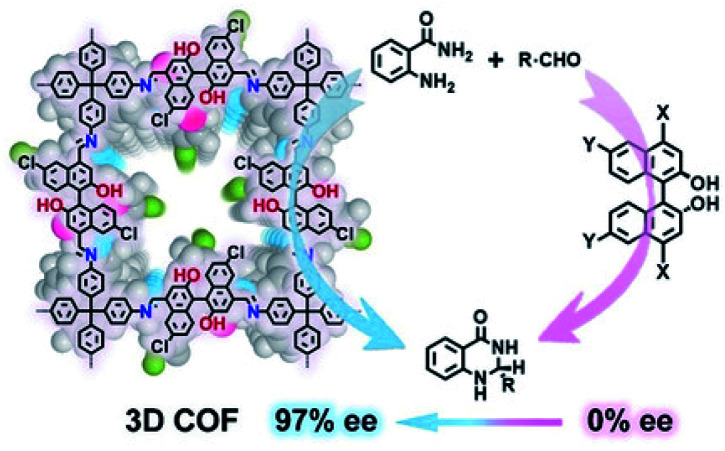
Confinement-driven enantioselectivity of the asymmetric acetalization promoted by 3D CCOFs. Adapted from ref. 15. Copyright 2021 Wiley.

Optically pure 1,1′-binaphthol (BINOL) derivatives are common privileged chiral ligands/catalysts for asymmetric synthesis. To improve enantioselectivity, a common approach involves introducing bulky substituents on the 3,3′-positions.^58^ However, this approach requires sophosticated catalyst synthesis and is often low-yielding. As such, an alternative approach that circumvents functionalising 3,3′-positions is highly desirable. Towards this end, Cui and Liu *et al.* introduced non-3,3′-substituted BINOL derivatives into CCOFs for asymmetric catalysis (Fig. 16).^15^CCOF 15 successfully promoted the asymmetric acetalisation of aminobenzamide and aldehydes with moderate to excellent enantioselectivities (71–97% e.e.) surpassing those of their homogeneous counterparts. Thus, this experiment demonstrates that the chiral cavities of the porous frameworks in combination with chiral catalysts are capable of stereocontrol of desired chiral products. Interestingly, the isostructural CCOF 16 afforded the desired products with a low e.e. opposite to the configuration as compared to CCOF 15 in the acetalisation of aminobenzamide, probably owing to the larger pores of CCOF 16. Large pores likely diminish chiral confinement effect, reduce enantioselectivity, and produce products with revsersed enantioselectivity. This reversed enantioselecitivity is similar to enzyme pocket dominates in enzymatic catalysis. In addition, CCOF 15 as a heterogeneous catalyst showed high recyclability and maintained activity and enantioselectivity of heterogeneous catalysts for 10 runs.

In 2019, Dong *et al.* reported that two CCOFs containing porphyrin and metal nanoparticles (M NP) as heterogeneous catalysts were employed in the thermally-driven asymmetric catalysis by transferring light energy into thermal energy under visible light irradiation. These CCOFs provided an eco-friendly strategy to enhance the yield and enantioselectivity of asymmetric transformations.^59^ Interestingly, Au@CCOF-CuTPP successfully promoted the asymmetric Henry reaction of benzyl alcohols and nitromethane in the excellent yields (93–99%) with satisfactory enantioselectivities (94–98% e.e.) under illumination with visible light. Meanwhile, Pd@CCOF-CuTPP smoothly catalyzed the asymmetric A^3^-coupling reactions to obtain the desired products with 68–98% yields and 90–98% e.e. through photothermal conversion. Additionally, the research team utilised (*R*)-CuTAPBN-COF as an asymmetric catalyst to achieve the synthesis of (*S*)–CIK in 98% yield with 94% e.e. *via* photothermal conversion under visible-light irradiation.^60^

Additionally, CCOFs constructed by chiral induction have a unique chiral microenvironment that can provide favourable conditions to realise asymmetric transformation. Cui and Liu *et al.* used the CCOFs induced by enantiopure 1-phenylethylamine to produce chiral Lewis acid catalysts through the coordination of enaminone and Cu(ii) ions.^30^ These chiral catalysts successfully promoted the asymmetric Henry reaction of nitroalkane and aldehydes with low catalytic activity and enantioselectivity, demonstrating the chiral channels are of great importance for asymmetric catalysis. Whereafter, the Dong group reported enantiopure DTP-COF as a chiral heterogeneous catalyst favourably catalyzed the asymmetric Michael addition reactions with high enantioselectivity, and allowed for the preparation of target products on the gram-scale level. Through this work, the Dong group demonstrated that the resultant β-ketoenamine-CCOFs could be populated with Cu(ii) ions to produce the Cu(ii)@CCOF catalysts for the asymmetric A^3^-coupling reaction with excellent yields and e.e. values.^47^

In summary, CCOFs as heterogeneous catalysts exhibited desirable potential to promote asymmetric transformations owing to the tunability of their structure and functionalities. Particularly, some CCOF-based catalysts exhibited better enantioselectivity than the corresponding homogeneous counterparts attributed to confinement effects exerted by the well-defined channels. Given the framework of COFs allows for the anchoring of privileged chiral ligands and organocatalysts, this property was exploited to achieve asymmetric catalysis through different synthetic approaches, however, only the limited catalytic system exhibited high reactivities and enantioselectivities in asymmetric transformations. It is still an enormous challenge to regulate the chiral environment and electronic effects of active sites in COFs to improve the activities and stereoselectivities in asymmetric catalysis. Specifically, the trade-off between asymmetry and crystallinity immensely restricts the construction of CCOFs with versatile functionality, this, in turn, makes synthesis more difficult and hinders the development and application of COOFs as heterogeneous catalysts. The synthesis of CCOFs is non-trivial and a research bottleneck exists in the synthesis of these materials. As such an emphasis needs to be placed on the synthesis and design of CCOFs for asymmetric catalysts with high activity and stereoselectivity.

### 4.2 Chiral separation

#### 4.2.1 Adsorptive separation

Adsorptive separation is an effective method for the enantiomeric separation of the analytes through the formation of the diastereomeric complexes based on specific host–guest interactions including hydrogen bonding, van der Waals forces, and electrostatic absorption. These interactions result in a difference in binding and releasing energy between the diastereomeric complexes, which has been extensively applied in the field of chiral separation. Mastai *et al*. in 2009 synthesised chiral mesoporous silica (CMS) sphere based on poly(ethylene oxide) and dl-glutamic acid [PEO113-*b*-(GluA)10] for the selective separation of valine enantiomers with an enantioselective factor of 5.22.^61^ Cui *et al*. employed enantiopure 1,10-biphenol derivates to construct a series of chiral MOFs as adsorbents for the high enantioselective separation of chiral analytes such as amines and mandelate derivatives, attributed to the different specific binding energies of the diastereomeric MOF–analyte complexes through the subject–object interactions in the microenvironment of the frameworks.^62^ Recently, Hu *et al*. reported the chiral porous organic polymer (CPOP) containing chiral 1,2-bis(3,4-dichloromaleimide)cyclohexane for the enantioselective adsorption of a range of racemic alcohols with up to 72% e.e., which further laid the foundation for chiral chromatography separation with high performance.^63^ Therefore, it is crucial to select adsorptive materials for enantioselective separation.

**Fig. 17:**
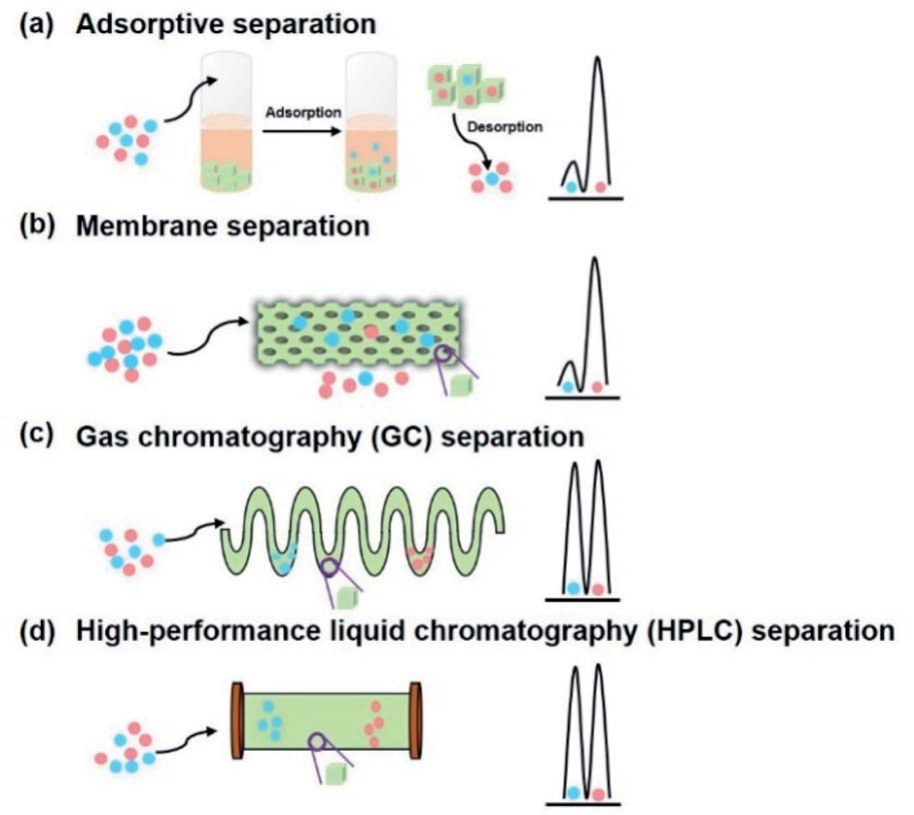
Schematic illustration of chiral separation on CCOFs: (a) adsorptive separation, (b) membrane separation, (c) gas chromatography (GC) separation, and (d) high-performance liquid chromatography (HPLC) separation. The green cubes represent CCOFs.

In the pursuit of an effective and rapid chiral adsorption and separation, the development of late-model adsorbents is particularly important for the application of adsorption technology. In comparison with conventional adsorption materials such as activated carbon and zeolite, COFs as advanced porous materials exhibit predominant separation performance due to their attractive superiorities including high porosity, well-defined channel, ultrahigh surface area, and facile functionalisation. Wang *et al.* explored the adsorption selectivity for CO_2_/N_2_ mixtures of 3D-Py-COF.^64^ Jiang *et al.* utilised TAPB-BMTTPA-COF as the adsorbent for removing toxic metal ions such as Hg(ii) and Pb(ii) in a highly selective manner.^65^ Beitle *et al.* reported Py-BPy^2+^-COF as an ion exchange material for biomolecule separation with high capacity and selectivity.^66^ Therefore, the framework of COFs contributes to various interactions including hydrogen bond interactions, π–π interactions, and electrostatic interactions, which endow the material with the ability of adsorptive separation. In consideration of the unique features of COFs, chiral selectors immobilised on COFs provided a chiral environment to explore functionalised CCOFs as chiral adsorbents for enantiomeric separation through the host–guest interactions (Fig. 17a).

**Fig. 18:**
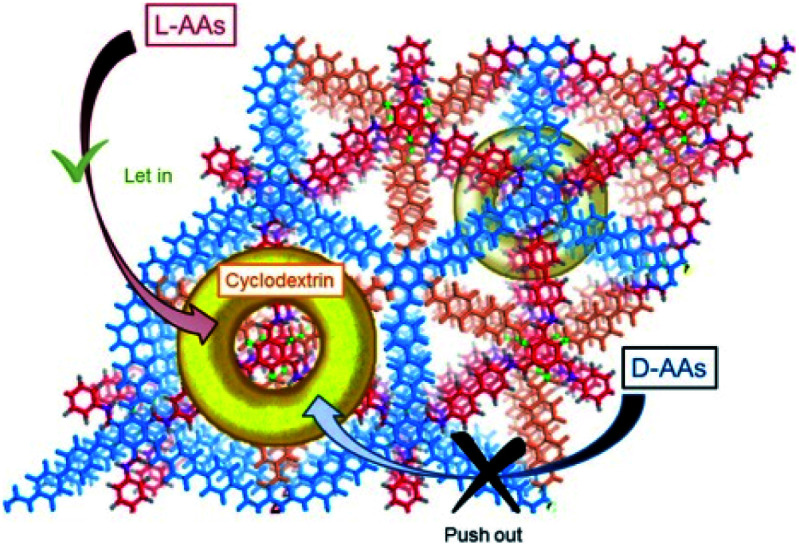
Schematic illustration of enantioselective adsorption of TpBD-Am7CD for amino acids. Adapted from ref. 29. Copyright 2021 the American Chemical Society.

Recently, Ji *et al.* incorporated chiral Am7CD into carboxyl-functionalised COF TpBD–3COOH to synthesise TpBD-Am7CD for the chiral adsorption of amino acids. Their findings showed that TpBD-Am7CD was useful for the enantioselective adsorption of amino acid enantiomers including histidine, tryptophan, phenylalanine, and tyrosine (Fig. 18).^29^ Moreover, the chiral TpBD-Am7CD displayed better enantioselectivity in comparison with the achiral TpBD–3COOH in the adsorption experiment. In addition, TpBD-Am7CD exhibited a similar chiral separation to that of the reported β-CD COF in adsorbing amino acid enantiomers, while the overall amount of Am7CD loaded onto TpBD-Am7CD was much less than that in β-CD COF. These results suggest that the synergy between the framework of the COFs and chiral selectors improves the chiral recognition abilities of the system as a whole.

The above studies demonstrate that COFs have the ability to achieve the separation of chiral analytes by adsorption. However, there are some problems that restrict the adsorptive separation of enantiomers. For instance, aperture size, specific surface areas, adsorptive force, and chiral recognition have a non-negligible influence on the adsorptive separation of COFs. Another considerable hurdle regarding the stability of COFs needs to be highlighted as it concerns one of the main principles in adsorptive separation and the movement of guest molecules. Some COFs are liable to change their framework or even collapse after the removal of guest molecules, thus decreasing the favourable framework interactions that endow CCOFs with such desirable enantioselectivity. Improving the stability of COFs is not only beneficial to enhancing the repeatability and durability of the adsorption materials, but also can expand the scope of application in adsorptive separation, especially under harsh conditions.

#### 4.2.2 Continuous-flow separation

Chiral chromatographic separations can utilise the enantiomers and the chiral stationary phases (CSPs) to produce a mixture of diastereomeric optical isomers, resulting in the enantioselective separation of the analytes attributed to the large physical differences between the diastereomers. These chiral chromatographic separations have been one of the most effective and extensive methods to achieve continuous separation of the chiral analytes *via* mobile phase elution. The chromatography techniques involve gas chromatography (GC), liquid chromatography (LC), supercritical fluid chromatography (SFC), and capillary electrochromatography (CEC), which have been intensively applied in numerous fields including chemical industry, agriculture, and pharmaceutics with excellent sensitivity and efficiency. Recently, Yang *et al.* reported nanosized porous organic cages (POCs) for chiral GC separation of chiral alcohols with good resolution and selectivity through the hydrogen-bonding and π–π interactions.^67^ Cui *et al.* synthesised the highly stable Zr-based MOFs with chiral crown ether moieties as CSPs for reversed-phase high-performance liquid chromatography (RP-HPLC), which achieved the enantioselective separation of amino acids and N-containing drugs with high resolution, selectivity, and durability.^68^ As the requirement for chiral compounds is increasing, the development of novel CSPs is of great significance.

For enantioselective separation with high selectivity and efficiency, the development of novel CSPs has been the focus of continuous flow separation. Recently, COFs as crystalline porous materials exhibited excellent performance in the field of chromatographic separation owing to controllable channel and facile functionalisation. For instance, Cui *et al.* reported 3D salen-based COFs as SPs for the HPLC separation of C8 alkylaromatic isomers, which demonstrated excellent efficiency and repeatability.^69^ The as-prepared column containing COF TFPB-BD as the SP displayed a remarkable separation of the analytes such as chlorobenzenes, alkylbenzenes, and phenolic compounds, with high efficiency and high resolution.^70^ To achieve selective separation of enantiomers, the chiral environment plays an essential role in the separation process. COFs can be constructed to possess chiral channels with an adjustable pore environment for the recognition of the chiral analytes through some specific interactions such as hydrogen bonding and electrostatic interactions. CCOFs as chiral stationary phases (CSPs) have the striking ability to efficiently separate enantiomers through the stereospecific formation of differentiable transient diastereomeric complexes in chiral resolution (Fig. 17). In consideration of the unique enantioselectivities of CCOFs for chiral molecules, recent progress in the enantiomeric separation of CCOFs as CSPs will be reviewed in this section.

**Fig. 19:**
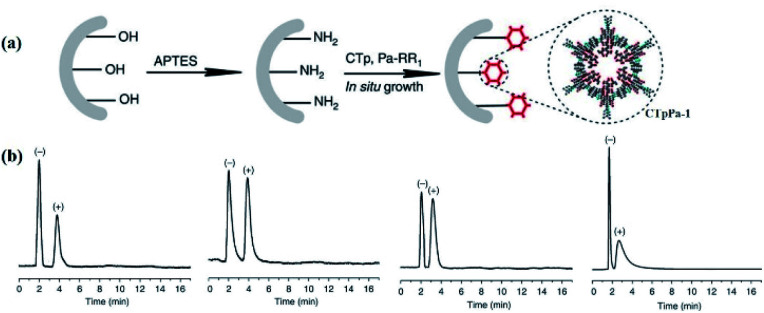
(a) *In situ* preparation of CTpPa-1-bound capillary columns. (b) Gas chromatograms of CTpPa-1-bound capillary column for 1-phenylethanol, 1-phenyl-1-propanol, limonene, and methyl lactate. Adapted from ref. 20. Copyright 2016 Nature Research.

In 2016, Yan *et al.* first developed an *in situ* growth method to afford CCOF-bound capillary columns for chiral gas chromatography.^20^ The obtained CCOF-columns were constructed from the functionalised CCOFs with (+)-diacetyl-l-tartaric and displayed high resolution for the chiral discrimination of enantiomers, including 1-phenyl ethanol, 1-phenyl-1-propanol, limonene, and methyl lactate. In addition, the prepared CCOF column showed excellent repeatability and recyclability in enantiomeric separation (Fig. 19). Notably, the obtained CCOF columns exhibited greater separation factors and better resolutions for 1-phenyl-1-propanol and limonene as compared to commercial chiral capillary columns. Moreover, the findings demonstrated that the (+)-enantiomers exhibited more order in the microenvironment of the CCOFs than the (−)-enantiomers, and the retention and chiral discrimination of enantiomers were facilitated by enthalpy.

**Fig. 20:**
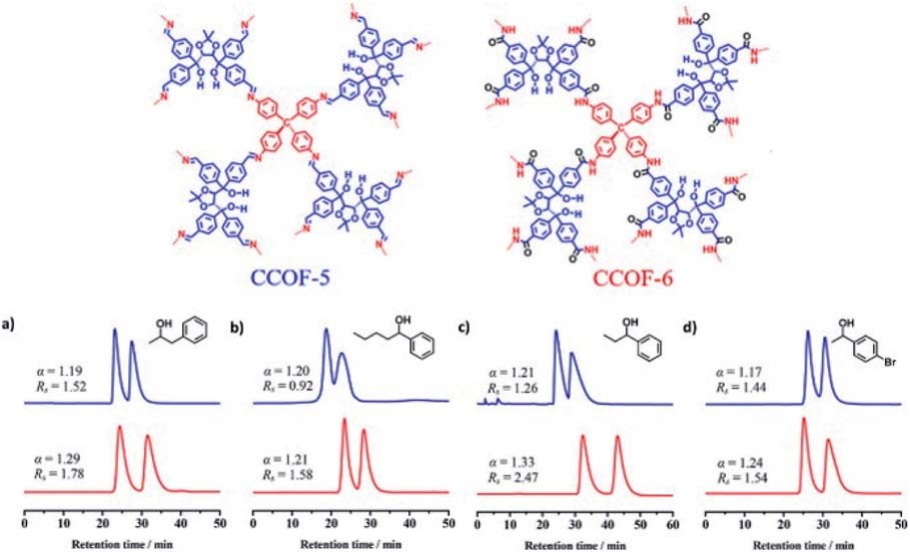
HPLC separation of CCOF-5 (blue line) and 6 (red line) packed columns for (a) 1-phenyl-2-propanol, (b) 1-phenyl-1-pentanol, (c) 1-phenyl-1-propanol and (d) 1-(4-bromophenyl)ethanol. Adapted from ref. 37. Copyright 2018 the American Chemical Society.

In 2018, Cui and Liu *et al.* exploited two 3D CCOFs with chiral TADDOL units to prepare an HPLC column for the separation of enantiomers *via* padding a mixture of the crystalline samples and silica (Fig. 20).^37^ The obtained columns successfully achieved the baseline separation of various racemic alcohols through the elution sequence of the *S*-enantiomer followed by the *R*-enantiomer. Interestingly, the resolution performance of the CCOF-6 column was superior to that of the CCOF-5 under similar conditions owing to hydrogen bonding interactions from the amide linkages on the CCOF-6, which favour the enantioselectivity during the adsorption process. Moreover, separation performances of the CCOF 6-based column remained for at least 2 months, suggesting substantial stability and repeatability of the column. The CCOF column could not, however, separate the 1-(1-naphthyl)-ethanol enantiomers, as the enantiomers are larger than the maximum channel sizes of the CCOFs. Meanwhile, the HPLC columns loaded with amorphous COF@SiO_2_ or (*R*, *R*)-TTA/SiO_2_ hybrid microspheres are incompetent in recognizing the discrimination of enantiomers. These findings confirm that the amphoteric chiral channels of CCOFs that load the chiral alcohol play a significant role in the chiral separation.

With the consideration of the enantiomer discrimination of biomolecules, Ma *et al.* immobilised biomolecules (such as lysozyme, tripeptide, and lysine) into achiral COFs to obtain CCOFs (biomolecules⊂COFs) for chiral separation. Compared to other biomolecules⊂COFs, the obtained lysozyme⊂COF 1 as CPS showed high chiral separation efficiency for various enantiomers such as tryptophan, leucine, and threonine, in both normal-phase and reverse-phase HPLC.^44^ The difference in chiral separation is probably influenced by the quantities of chiral centres, structural complexity, and amphipathicity of biomolecules, which was verified by investigating the chiral separation capacities of biomolecule⊂COF 1. In addition, the HPLC-column loading lysozyme⊂COF 1 performed 120 separation runs with continual high efficiency over two months, indicating the high reusability and durability of lysozyme⊂COF 1.

**Fig. 21:**
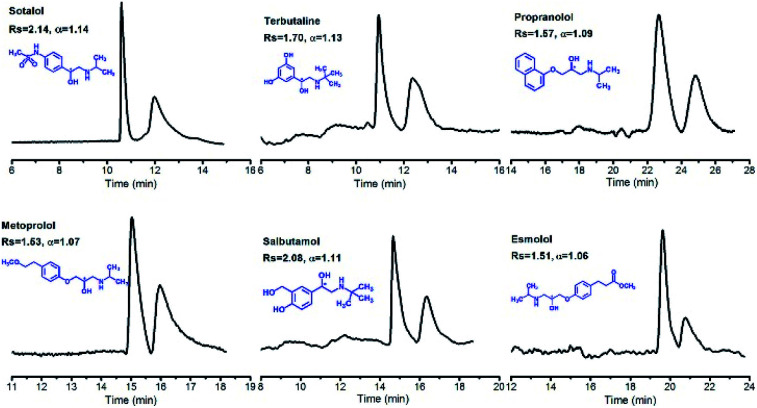
Resolution of the β-CD COF-packed capillary electrochromatography for enantiomers on samples, including sotalol, terbutaline, propranolol, metoprolol, salbutamol, and esmolol. Adapted from ref. 71. Copyright 2019 the American Chemical Society.

Recently, Ji *et al.* anchored the as-synthesised β-CD COF on the inner wall of a capillary *via* photopolymerization to fabricate CECs for chiral separation of enantiomeric drugs (Fig. 21).^71^ These capillary columns exhibited superior enantioselectivity and high resolution, and achieved baseline separation of drug enantiomers such as sotalol, terbutaline, and propranolol. The outstanding separation efficiency of the obtained column with β-CD COF is mainly attributed to the unique microenvironment of β-CD COF and the hydrophobic interactions between the analytes and CSP. Moreover, relative standard deviations (RSD%) of retention times and resolution of (±)-sotalol confirmed satisfactory stability and repeatability of the β-CD COF capillary.

**Fig. 22:**
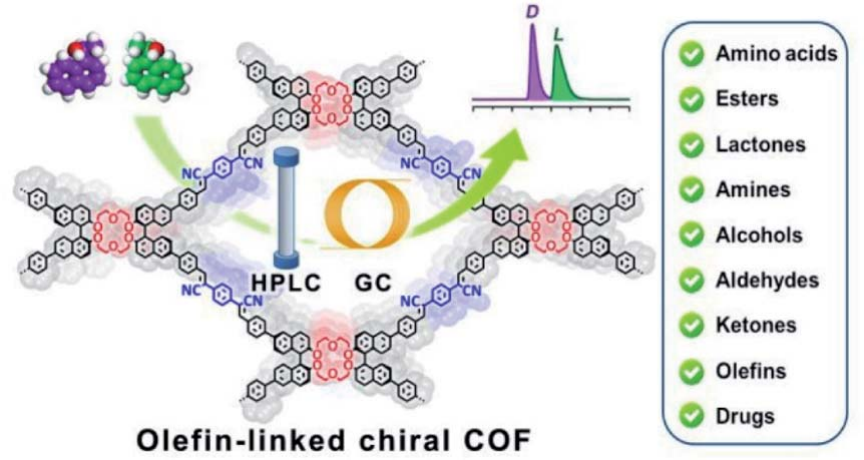
Schematic illustration of COF-packed HPLC and GC on the olefin-linked CCOFs packed columns. Adapted from ref. 72. Copyright 2022 the American Chemical Society.

The weak stability of imine-based COFs ultimately restricts their applications as CSPs, so to address this, Cui and Yuan *et al.* employed the olefin-linked CCOFs, which are highly stable under harsh conditions, to fabricate the CSP packed columns for GC and normal-phase (NP) and reversed-phase (RP) HPLC (Fig. 22).^72^ Based on the unique identification ability of the chiral crown ether sites and the pore confinement of frameworks, the CCOF CSP-packed column demonstrated separation of the amino acid enantiomers with high enantioselectivity and good resolution under RP solvent systems, while achieving the effective segregation of other chiral compounds and drugs under NP chromatographic condition through intermolecular interactions. Moreover, the COF-packed GC columns effectively completed the enantioseparation of various racemes with flying colors. The research demonstrated that the separation effects of olefin-linked CCOFs as CSPs were influenced by the shape and particle size of the obtained CSPs, in addition to the pore size effect of CCOFs. For instance, CSP-1 packed column with CCOF 17 for HPLC demonstrated the excellent enantioselectivity and the good resolution ability during the separation of some racemates, while the CSP-3 packed column with CCOF 17 could not selectively separate any analytes due to the irregular shape and nonuniform particle size of the CSP. The chromatographic column with CCOF 17 showed superior separation for some smaller racemates, while the column with CCOF 18 showed more satisfactory separation for some larger racemates, indicating the dimension matching for the substrates. Interestingly, the size effect of the CCOF channel made for the excellent complementarity of the chromatographic columns modified by CCOF 17 and 18 for the separation of numerous racemates, including amino acids, aldehydes, amines, alcohols, lactones, esters, and olefins. Moreover, the mobile phase with K^+^ was found to inhibit the chiral recognition for HPLC, due to a strong interaction of the crown ether moieties with K^+^. Notably, the selectivity and durability of the resulting columns all rival that of related commercial chiral columns, while the versatility of CCOF-based columns is superior to other chiral columns. However, it is difficult to accurately elaborate on the interaction mechanism of the enantioseparation by CSPs. The crown ether groups and the channel microenvironment of the olefin-linked CCOFs may play a crucial part in the enantioselectivity and chiral recognition ability of racemates. Firstly, the unique configuration of crown ethers results in the generation of crown ether and analyte complexes through hydrogen bonding and host–guest interactions. Besides, the π–π interactions of chiral compounds and CSPs may also participate in the enantioseparation of racemates, other interaction forces including hydrophobic, dispersion, and dipole–dipole may also affect the enantioselectivity during chiral separation.

Examples of CCOFs as CSPs summarised have demonstrated that CCOFs, especially those with ultrastable olefin linkages, have a remarkable capacity to achieve the enantioselective separation of racemes in chromatographic resolution, and show potential application foreground for the separation of chiral drugs. Although the synthesis and application of CCOFs as CSPs have made some progress, they still face tremendous challenges for enantioseparation in chromatographic resolution. Firstly, it is necessary to improve the stabilities of CCOFs as CSPs. Currently, most of the available CCOFs as CSPs are limited to C=N bonds which display weak chemical stabilities, thus impeding the further application of chromatographic fractionation in harsh chemical conditions such as strong acid or base conditions. This issue may be responsible for limiting the CCOF-packed columns for reversed-phase HPLC with high resolution and selectivity. The poor stabilities of CSPs could additionally result in the collapse of the framework during enantioseparation which would be harmful to the repeatability and durability of the obtained chromatographic column without obvious loss of separation efficiencies. The reported findings indicate that the chiral separation of chromatography mainly rests with not only host–guest interactions including π–π interactions, hydrogen bonding, and *van der* Waals forces, but also a steric hindrance, and the size and shape of well-defined channels. These variables are essential to be explored further through the combination of experiment and theoretical calculation. Therefore, the separation mechanism of CCOFs as CSPs should have an in-depth study to provide the theoretical guidance for the design and construction of novel CCOFs with exceptional separation effects.

#### 4.2.3 Membrane separation

Membrane separation is an efficient enantioselective separation approach that has drawn dramatic attention in the pharmaceutical industry owing to its conspicuous superiority including low operating cost, high-efficiency resolution, ease of operation, and low energy consumption. Enantioselective membranes act as barriers that selectively transport one enantiomer in the resolution process. This occurs because the formation of diastereoisomeric complexes causes a difference in Gibbs free energy and stability between the enantiomer and chiral recognition sites resulting in the two enantiomers passing through the chiral membrane at different rates. In 2017, Lee *et al.* employed α-helical peptide self-assemblies to anchor into vesicular structures to obtain enantioselective membranes which showed selective diffusion across the chiral peptide membrane.^73^ Wang *et al.* recently developed chiral MOF mixed matrix membranes (MMMs) with amino acids to selectively separate 1-phenylethanol enantiomers up to 100% e.e.^74^ In addition, Nono-Tagne *et al.* fabricated a cellulose tris(3,5-dimethylphenylcarbamate) (CDMPC) membrane with commercial polytetrafluoroethylene filters for the chiral separation of (*R*, *S*)-1-(1-naphthyl)ethanol to afford the *S*-enantiomer with 32.9% e.e. through hydrogen bonds and π–π stacking interactions.^75^ With increasing requirements for rapid and high-throughput enantiomer separation, chiral membrane separation showed promising abilities to efficiently achieve enantiomeric resolution of the racemates through the differences in interactions between chiral recognition sites and different enantiomers under the action of external forces including osmotic pressure, voltage, and pH gradients. However, high selectivity is still an intractable issue for chiral separation of conventional membranes as a consequence of the limit of permeability. With the development of emerging membranes based on advanced porous materials,^73,76^ COFs are a promising platform for membrane separation, with the ability to balance the relationship between permeability and selectivity to achieve efficient and effective separation of enantiomers by regulating the structure and function of CCOFs (Fig. 17b).

Cui *et al.* exploited the homogeneous suspension of as-prepared CD-COFs and poly(ether sulfone) (PES) to construct flexible MMMs for the enantioselective transport of amino acids by the phase-inversion method.^45^ SEM revealed that the surface of the as-prepared MMMs is evenly distributed with CD-COF particles, which favourably secures selective transport of amino acids through nanochannels of the CD-COFs. The separation factors rl-His/rd-His are 34.0 for the CD-COF-1 MMM and 1.7 for the CD-COF-2 MMM, respectively, suggesting higher enantioselectivity of the CD-COF-1 MMM for l-His as compared to the CD-COF-2 MMM due to the difference of contact angles and surface zeta potentials on the obtained membranes. Again, transmembrane permeation tests also demonstrated the CD-COF-1 MMM penetrated l-His much faster than d-His, probably due to the synergistic effect between the osmotic pressure difference and enantioselective adsorption and desorption. This work lays a foundation for the development of novel COF-based membranes to selectively transport chiral biomolecules.

**Fig. 23:**
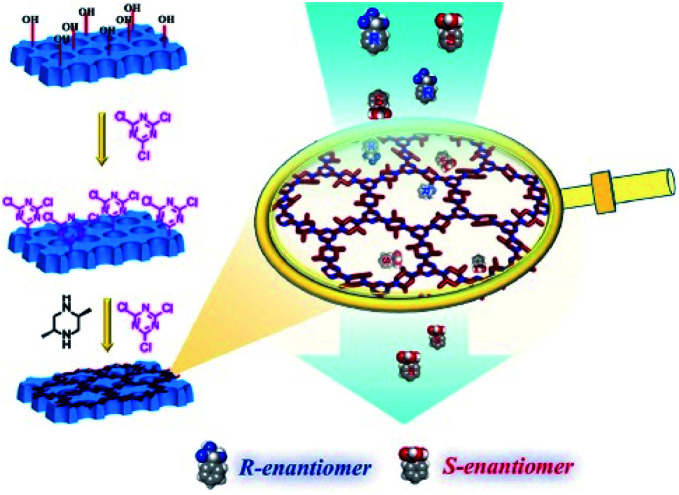
Schematic illustration of the fabrication and application of the CCTF membranes. Adapted from ref. 77. Copyright 2021 Elsevier.

Hu *et al.* demonstrated that novel chiral covalent triazine framework (CCTF) membranes based on porous quartz substrate fiber membranes were constructed through the ‘‘*in situ* growth’’ method, and applied in the chiral separation of drug enantiomers (Fig. 23).^77^ CCTF membranes exhibited good enantioselectivity for the analytes, including 1-phenylethanol, 1,1-binaphthol, and ibuprofen, by the synergistic interaction between unique chiral channel structures and hydrogen bonds. Again, CC-DMP CCTF displayed better enantioselectivity for chiral drugs as compared to CC-MP CCTF under the same conditions due to the difference in the adsorption energy and the dimension matching for the enantiomers between CC-DMP CCTF and CC-MP CCTF. For instance, the CC-DMP CCTF displays higher adsorption energy for the (*S*)-1-phenylethanol as compared to CC-MP CCTF, demonstrating that CC-DMP CCTF facilitates the enantioselective separation of enantiomers. CC-DMP CCTF has chiral channels with larger aperture sizes and thus exhibits the preferential dimension matching for racemates to achieve effective separation. These results further indicate that the separation effect of CCTFs is in connection with the quantities of the chiral centres. Furthermore, the mechanism of enantiomer recognition and separation was confirmed by quantum mechanical calculations and liquid chromatography.

Although the CCOF-based membranes have exhibited potential advantages in enantioselective separation, suggesting the feasibility for chiral separation of enantiomers, the CCOF-membranes coexist with great opportunities yet tough challenges. For a chiral separation membrane, the ideal separation effect should simultaneously hold high enantioselectivity and high permeability, which usually require a membrane with a dense and uniform channel. However, the insolubility of CCOF-membranes limits their ability for the solution-based processes that allow for defect-free membranes, further impeding the CCOF-membranes' application in chiral separation. Although the MMMs are able to integrate the selectivity of CCOFs and the superiorities of polymer matrices, thus bypassing the typical “trade-off” relationship between selectivity and permeability, it is difficult to accurately control the formation process of the membranes with CCOF-fillers. The chiral separation effect of CCOF-MMMs is overall weakened by the maldistribution of CCOF-MMMs and the disorder of polymer chain segments. In addition, the separation performance of MMMs is also limited by the crystallinity and particle size of a COF powder. In contrast, *in situ* polymerization can result in continuous and self-reliant CCOF-membranes with good crystallinity and regular channels, and thus avoid the blockage of the membrane pores. Despite overcoming the weakness of MMMs, the *in situ*-growth method suffers from difficulty in regulating the thickness of membranes and from the harsh conditions required for membrane preparation, both of which seriously hinder the chiral separation of CCOF-membrane. Therefore, there is a necessity to develop a preparation method for CCOF-membranes to achieve chiral separation of enantiomers with high selectivity and permeability.

### 4.3 Enantioselective sensing

#### 4.3.1 Fluorescence sensing

Fluorescence sensing has become an appreciable methodology, which has been paid substantial attention by researchers due to its dominant characteristics involving simplicity, high sensitivity, low cost, real-time analysis, and easy combination with other optical signals.^78^ Fluorescence chiral sensing plays a significant role in detecting chiral analytes, as subject–object interactions result in differences in fluorescence responses which causes diastereomeric sensor–analyst complexes to form. For instance, Pu *et al.* constructed a perfluoroalkyl-BINOL-based chiral diketone for the discrimination of amino alcohol enantiomers through host–guest interactions generating obviously enantioselective fluorescent enhancements.^79^ Zheng *et al.* reported a neutral chiral receptor containing TPE cyclohexylbisurea for the chiral recognition of enantiomers including acidic compounds, basic compounds, amino acids, and even neutral alcohols with high selectivity and sensitivity through the multiple hydrogen bonds and CH–π interactions between the TPE urea receptor and the enantiomers.^80^ Recently, Cui *et al.* developed a chiral Eu-MOF-MMM for the enantioselective fluorescence sensing of both terpenes and terpenoids through the formation of diastereoisomeric complexes between the analytes and chiral phosphoric acid active sites.^81^ Therefore, it is of great significance for the development of chiral sensors with fluorescence for the detection of chiral analytes especially, chiral pharmaceuticals. This research has overall demonstrated a novel synthetic strategy for the construction of an enantioselective fluorescent receptor that features heterotropic positive pseudo-allosterism. This type of receptor design could be useful in the development of sensors and materials that can be used in facilitated transport or molecular mixture separations.

While fluorescent COFs as chemical sensors have previously depended on π-conjugated organic construction units to display strong fluorescence emission, recent COF chemical sensors have exhibited outstanding advantages in the field of fluorescence recognition owing to their features of permanent porosity, optical tenability, and fluorescence diversity.^82^ Numerous fluorescent COFs have thus far been widely employed in fluorescence sensing of metallic ions, organic solvents, humidity, pH detection, and biosensing.^83^ Notably, progress has also been made in associating chiral structural units and fluorescence units to construct fluorescence CCOFs. These CCOFs exhibit the capability of enantioselective recognition of chiral compounds to evaluate the absolute configuration of enantiomers through chiral host–guest interaction and optical responses.^24,30,38^

**Fig. 24:**
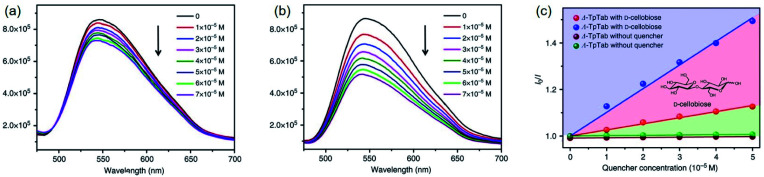
(a and b) The fluorescence emission spectra of the (Λ)/(Δ)-TpTab COF with increasing concentration of the d-cellobiose quencher. (c) SV plots of the fluorescence emissions of the (Λ)/(Δ)-TpTab COF quenched by d-cellobiose. Adapted from ref. 30. Copyright 2018 Nature Research.

In 2018, Cui and co-workers reported that CCOF-TpTab with intense fluorescence emissions was utilised as a chiral fluorescence sensor for enantioselective sensing of saccharides through the interaction between the saccharides and the enantiomers of TpTab.^30^ When treated with aliquots of d-saccharides such as d-cellobiose, the CCOF's fluorescence emission at 540 nm was quenched due to the formation of a host–guest complex. The decrease in fluorescence rate was greater with (Λ)-TpTab than with (Δ)-TpTab, indicating enantioselectivity in the fluorescent recognition (Fig. 24). Of note, the quenching ratio (QR) values of the tested saccharides are comparable to those of sugar derivatives examined by fluorescence sensors of the binol-bisboronic-acid.

**Fig. 25:**
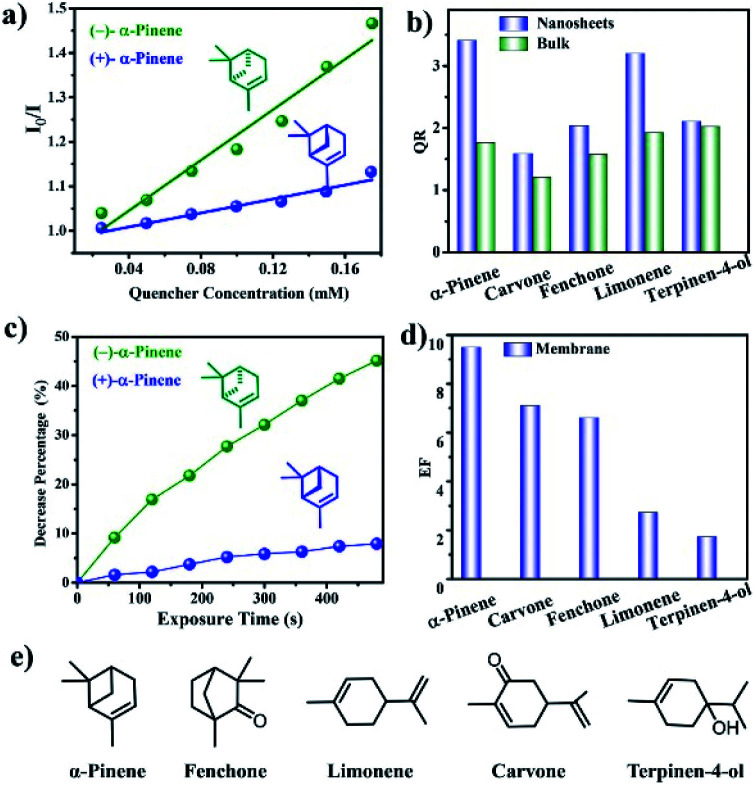
(a) Stern–Völmer plots of 7-NS upon titration of α-pinene in MeCN. (b) Enantioselective quenching ratio for several terpenes. (c) Decrease percentage upon exposure to α-pinene for 7@PVDF (d) Enantioselective fluorescence decrease ratio for several vapours. (e) Chemical structures of terpenes were used in the study. Adapted from ref. 38. Copyright 2019 the American Chemical Society.

BINOL derivatives as versatile fluorophores have been extensively employed in chiral fluorescence sensors such as organic oligomers and polymers for chiral recognition, by way of hydrogen-bonding interactions between the detected analyte and BINOL units. This gives them unique characteristics, including special chiral and aromatic structures, high luminaire efficiency, and facile functionalisation. In 2019, Cui *et al.* incorporated the BINOL skeleton into CCOFs with the confinement effect and the conformational rigidity to assess the enantioselectivity of chiral vapours. The obtained CCOF-7 could show intense fluorescence related to construction units.^38^ This COF contains flexible TPE moieties that could be readily stripped into ultrathin nanosheets, named 7-NS, with high fluorescence emission different from that of bulk material, caused by the reduction of interlayer π–π interactions in nanosheets. In contrast with CCOF-7, 7-NS possessed more accessible active binding sites on the external surface to contact and interact with the analyte, and also exhibited superior sensitivity and enantioselectivity for chiral vapours including pinene, limonene, fenchone, carvone, and terpinen-4-ol (Fig. 25). In order to overcome clogging or recycling issues in practical applications, the 7-NS/PVDF solution was electrospun onto aluminum foil substrates to produce freestanding nanofiber membranes, 7@PVDF, which demonstrated the favourable capability of chiral fluorescence recognition for terpene vapours. The 7@PVDF membrane could be directly reused for at least 3 runs of fluorescent recognition without obvious loss of sensitivity and enantioselectivity. Interestingly, the findings illustrated that the prepared CCOF NSs show conspicuous sensitivity to vapour quenchers in both the solution and the membrane with superior enantioselectivity in comparison to the corresponding BINOL-based system, which is currently ascribed to the confinement effect of the CCOF channels and conformational rigidity of the fixed BINOL units. The research provides an attractive strategy for overcoming unobvious structure change in chromophore moieties to recognise chiral vapour discrimination by optical methods.

Inspired by crown ethers for the chiral recognition of organic cations, the Cui group constructed the C=C bond-linked CCOFs with a chiral crown ether skeleton by Knoevenagel polycondensation, and obtained C–C single bond-based CCOFs by reduction of olefin-linked frameworks.^24^ Compared to the parent frameworks, the reduced CCOFs exhibited blue-shifted emission with increased quantum yields, possibly attributed to the formation of C–C bond linkages hindering π–π interactions between COF layers in addition to hindering the non-radiation energy transfer. However, the olefin-linked CCOFs as fluorescent sensors for amino alcohols revealed superior enantioselectivity through hydrogen bonds between the N^+^–H bonds and the crown ethers, as compared to the reduced CCOFs.

Although some progress has been made in chiral fluorescence sensors, COFs are still a new member of fluorescent probes, meaning there is an enormous challenge in constructing fluorescence CCOFs for chiral recognition of enantiomers with satisfactory sensibility and enantioselectivity. Currently, fluorescence sensing COFs mainly focus on the “turn-off” fluorescence sensor, however, the COFs suffer from photobleaching and consequently the accuracy of fluorescent recognition is reduced. Therefore, it is crucial to develop more sensitive and precise fluorescence sensors like the ratio sensor, which has a capacity for internal self-calibration to eliminate the interference of other factors for accurate recognition of the analytes through the ratio of fluorescence intensity along with the linear change in analyte concentration. In addition, the stabilities of fluorescence COFs are also worthy of consideration. Because fluorescence probes may become fragile, this would impair the recognition of analytes in a physiological environment over an extended period. Another crucial element of a chiral sensing system is the introduction of chiral selectors, which ensure the obtained fluorescence CCOFs show remarkable potential in chiral recognition.

#### 4.3.2 Electrochemical sensing

Electrochemical chiral sensing could reveal the fascinating capacity of enantioselective recognition based on the combination of electrochemical sensors and chirality recognition, thus it has attracted extensive attention in precision medicine, environmental analysis, food science, and bioresearch. The principle of chiral recognition is that the formation of diastereomeric complexes between chiral sensors and chiral analytes contribute to the change of electrochemical responses, including electrical current, potential, and resistance, to realise the chiral detection of enantiomers with high sensitivity and selectivity. For example, Liu *et al.* developed a graphene chiral sensor modified with acetylcholinesterase (AChE) for the discrimination of (+)/(−)-methamidophos with high selectivity and rapid off-line detection.^84^ Cheng *et al.* successfully achieved the discrimination of pinene enantiomers with high stability and sensitivity *via* an electrochemical method based on the chiral CD-MOF as an electrochemical sensor.^85^ Although diversified electrochemical assay has been developed, these methods still need to be improved in sensitivity and cannot always satisfy the requirement of *in vivo* metabolite probing and drug development. Accordingly, a novel chiral discrimination platform capable of simple operation, quick response, high sensitivity, and splendid selective detection of the weak chiral signal is highly desired in various applications.

COFs as crystalline porous polymers could display numerous advantages such as high π-conductivity and facile functionalisation, which have shown great potential applications in electrochemical sensors through the functionalisation of electroactive monomers for improving analytical effect. For instance, Yang *et al.* developed a COF for the highly sensitive detection of cardiac troponin I.^86^ Wang *et al.* proposed COFs with ferrocence as ratiometric electrochemical sensors for the detection of H_2_O_2_.^87^ COFs also provide their advantages within chiral sensing, and have demonstrated their ability to discriminate enantiomers through the different electrical responses of the diastereoisomeric complexes between the CCOF sensors and the analytes.

**Fig. 26:**
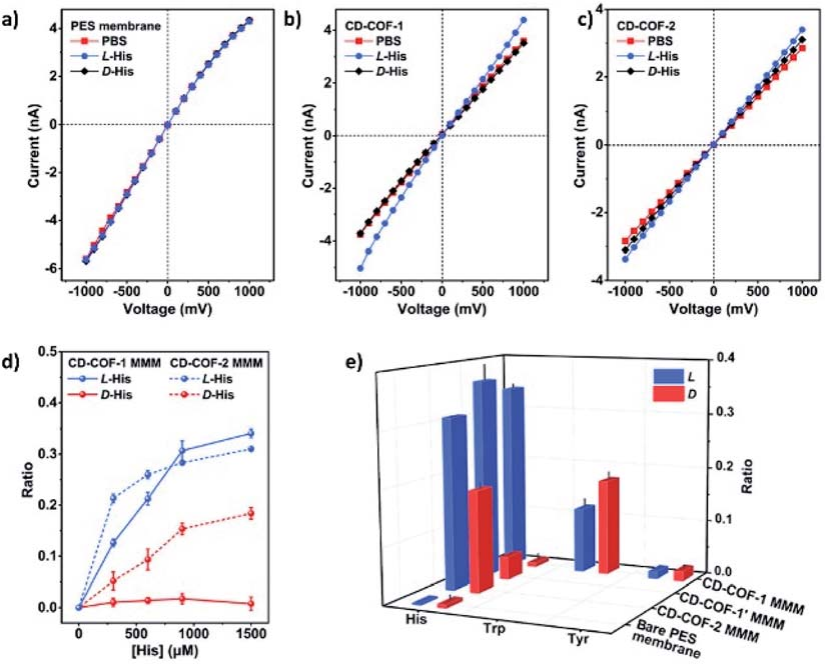
(a and b) *I*–*V* curves for the CD-COF MMMs in phosphate-buffered saline without or with the addition of l- or d-His. (c) *I*–*V* characteristics of the CD–COF–1 MMM in 20 mM PBS before and after addition of l/d-His. (d) Effect of increasing concentrations of His on the ionic current of the CD-COF MMMs. (e) Comparison of current change ratios for the CD-COF MMMs and bare PES membrane upon addition of amino acid enantiomers. Adapted from ref. 45. Copyright 2019 the American Chemical Society.

Inspired by chiral recognition based on cyclodextrin, Cui and co-workers developed a highly enantioselective method in which CCOF modified chiral β-cyclodextrins (β-CD) were fabricated into independent mixed matrix membranes (MMMs) for selective transport of amino acids by measuring ionic current signatures and concentration changes of osmotic analytes (Fig. 26).^45^ The transmembrane ionic current findings elucidated that the ionic current obviously increased with increasing concentration of l-His in the electrolyte, while the current remained unchanged with the change of concentration of d-His, implying the chiral recognition of CD-CCOF MMMs for the His enantiomers. This phenomenon is attributed to the selective binding of His enantiomers to active sites of CCOF channels, giving rise to a remarkable change in the transmembrane ionic current. Furthermore, the AA stacked CD-COF-1 MMM exhibited preferable stereoselectivity to l-His than the AB stacked CD-COF-2 MMM through the electronic signal, which is converted by excimer formation of the COF MMM and l-His, ascribed to its uniform open channels. This work indicated that functionalised CCOFs as electrochemical sensors have great potential and advantages in the selective transport of small molecules and even biomolecules at the nanoscale.

The application of electrochemical sensing for chiral recognition has been a research focus in the field of sensor technologies because chiral substances have incomparable importance in bromatology, pesticide areas, and bioscience. Currently, multitudinous electroactive materials and homochiral building units have been desired candidates for the construction of electrochemical sensing with the sensibility and stereoselectivity that are required for the electrochemical response of chiral recognition. In general, the use of CCOFs as new-style electrochemical sensors for chiral recognition is in its infancy. With reported research in mind, it is necessary to develop novel CCOFs for realising high sensibility and selectivity of chiral recognition in different fields through a judicious choice of electroactive building blocks and chiral molecules including crown ether, and chitosan. Additionally, the mechanism of chiral discrimination deserves attention in chiral electrochemical sensing, which is beneficial in guiding the design of sensing materials with impressive properties.

## Conclusions

CCOFs are emerging advanced materials with easy functionalisation and precision tunability, which show a promising potential for chiral expressions such as asymmetric catalysis, chiroptical property, and enantioselective separation. While the development of CCOFs is still in its infancy, they have demonstrated significant achievement over the past decade. In this review, we summarised the research progress of CCOFs including the synthetic strategies, chiroptical characteristics, and enantioselective applications in asymmetric catalysis, chiral separation, and enantioselective recognition. The reported methods ingeniously introduced chirality into the framework of COFs to greatly promote the development of CCOFs, however, there are some problems with these methods. Direct synthesis may contribute to racemization production or CCOF decomposition during their preparation under harsh conditions. Chiral sites are also not evenly distributed in the framework by PSM. In addition, the absolute configuration of CCOFs is mainly measured by ECD and CPL, however, ECD is limited by the relationship between chromophores and chiral centres, which is not conducive to analysing chiral information of frameworks in the ground state. It is consequently necessary to use Vibrational Circular Dichroism (VCD) and Raman Optical Activity (ROA) to further study the chiral information of CCOFs. Meanwhile, the construction of CPL-based materials, especially CCOFs with high quantum yields and *g*_lum_, is hampered by the relationship between fluorescence and chirality. Again, the stability of CCOFs has always been a stumbling block in the application of materials. For instance, both the recyclability of heterogeneous catalysts and the durability of CSP are subject to the stability of CCOFs. Therefore, it is indispensable to introduce stable linkages^88^ such as oxazoles, thiazoles, dioxins, and pyrazines to enhance the stabilities of CCOFs.

Stereocontrol of the desired products for chiral catalysts can be an intractable problem in the field of asymmetric catalysis. CCOFs have shown prominent promise as asymmetric catalysts due to their advantages such as tunability of well-defined channels and functionality. Nevertheless, the fabrication of CCOF-based catalysts remains in the preliminary stage. Hence, it will be an important task for the development of novel CCOF catalysts by the reasonable implantation of privileged chiral catalysts or ligands into the frameworks. Notably, Cui *et al.* utilised the direct synthesis to obtain Multivariate CCOFs.^35^ In addition, they realised asymmetric photocatalysis by combining achiral COFs as photocatalysts with chiral organic catalysts.^12*b*^ Again, Dong *et al.* artfully combined chirality and photoactivity to synthesise multifunctional CCOFs promoting asymmetric photothermal catalysis.^59^ The above findings indicate COFs are a promising platform to construct the multifunctional synergistic catalysts which will be in favour of promoting novel asymmetric catalysis including cooperative catalysis and tandem/sequential catalysis. Moreover, the theoretical calculation is of great significance to further elucidate the catalytic mechanism of CCOF heterogeneous catalysts. Cui *et al.* explained (*R*)-CCOF 15 gave (*S*)-2,3-dihydroquinazolinone (DHQZ) with higher stereoselectivity as compared to (*R*)-BDA in the asymmetric acetalization of 2-aminobenzamides with aldehydes by the density functional theory (DFT) calculations.^15^ Therefore, it is necessary to elucidate the mechanism in combination with DFT in-depth, which will make for the regulation of catalytic performance of CCOF catalysts. Furthermore, the reported findings indicate that the chiral separation mainly rests with not only host–guest interactions including π–π interactions, hydrogen bonding, and van der Waals forces, but also a steric hindrance, and the size and shape of well-defined channels, which is also essential to be explored further through the combination of experiment and theoretical calculation. Meanwhile, the novel functional molecules should also be concerned with expanding the scope of application in enantioselective separation, especially efficient chiral separation of biological macromolecules through the development of functionalised CCOFs and their composite material with other separating materials. In consideration of the importance of sensitive selectivity for chiral sensing, the central issue associated with chiral recognition of CCOFs is sensitivity based on differential interactions between probes and enantiopure guests. Although the ingenious combination of chirality and functional groups in CCOFs demonstrates unique enantioselective recognition for racemic analytes, the potential of CCOFs as chiral sensors is not completely demonstrated, attributed to the deficiency of design principles and chiral systems. In summary, opportunities and challenges coexist in the field of CCOFs, where a variety of functionalised CCOFs with fascinating proprieties will be developed by the regulation of structure and functionality in the future.

## Author contributions

The manuscript was written through the contributions of all authors. All authors have given approval to the final version of the manuscript.

## Conflicts of interest

There are no conflicts to declare.

## Supplementary Material
